# Film Recovery Constraints on Optical and Gravimetric Assessment of Pulsed Gas–Solid Cleaning of Model Plastic Films

**DOI:** 10.3390/polym18202593

**Published:** 2026-10-21

**Authors:** Chaojie Yu, Junqing Xu, Xichao Hu, Haochen Zhu, Guangming Li

**Affiliations:** 1 College of Environmental Science and Engineering, Tongji University, Shanghai 200092, China; 2030524@tongji.edu.cn (C.Y.); xjq0512@tongji.edu.cn (J.X.); haochen_zhu@tongji.edu.cn (H.Z.); 2 Shanghai Tianqiang Environmental Technology Co., Ltd., Shanghai 201415, China; 19117171706@163.com

**Keywords:** plastic film cleaning, gas–solid fluidization, optical recovery, apparent gravimetric change, film count recovery, measurement validity

## Abstract

Cleaning-induced mass changes depend on which films enter the post-cleaning weighing. This study quantifies that dependence using 84 model film runs in an established pulsed gas–solid apparatus and a laboratory stirred water reference. The count-matched weighing criterion retained 26 runs (31.0%): 24.0% of dry cleaning runs and 88.9% of stirred water reference runs. Ten of 21 groups had no eligible gravimetric observation. In one incomplete recovery run, assigning the mean initial clean film mass to the unweighed piece gave a scenario contribution of 19.5 percentage points of the unrestricted gravimetric index, illustrating potential bias without correcting the observation. Optical normalization denominators ranged from 68.0 to 76.2 percentage points; separately selected films introduced a conditional sampling uncertainty component of 0.33–0.99 percentage points in optical recovery. At 120 s, the nominal 5 g polyethylene (PE) configuration had 15.2 percentage point lower mean optical recovery than the 3 g configuration despite similar film count recovery. Reporting recovery-dependent coverage alongside each endpoint keeps laboratory comparisons tied to the film populations actually measured.

## 1. Introduction

Flexible plastic waste often requires cleaning before mechanical recycling. Waste source and contamination affect pretreatment requirements [1,2,3], while non-polymeric impurities [4], multilayer structures [5] and product design [6] constrain the recovered material. Impurities and composition affect the properties of recycled polyethylene (PE) and polypropylene (PP) [7,8], and printing components can alter recycled film properties [9]. Recyclate quality therefore involves several distinct measurements [10]. Optical transmission depends on film structure and surface condition [11], and polymer particles have been detected in recycling wastewater [12]. A reduction in the mass of recovered material can include both removed contamination and polymer absent from the weighed sample. Relating that reduction to cleaning requires a defined weighing population.

Studies of flexible-PE washing have examined washing conditions, melt processing and recovered material properties [13,14], as well as pretreatment and wash water reuse [15]. Deinking depends on agitation, liquid-to-solid ratio and ink resin chemistry [16,17]. Odorous compounds vary with collection source [18] and respond differently to washing media [19,20]. Ultrasonic cleaning studies have combined methods to characterize organic and inorganic contamination [21], while studies of food-contaminated packaging have examined both recovered plastic and separated residues [22]. These measurements address different components of a cleaning process. In a film experiment, even a high apparent mass change can become difficult to interpret if post-cleaning weighing excludes whole input pieces.

Gas–solid cleaning has been investigated for contaminated waste plastics [23], landfill-recovered plastics [24] and plastic lunchboxes [25]. Particle resuspension research considers fluid forces and surface adhesion [26], while pulsed fluidization and air jet studies describe bed behavior and particle removal under their respective conditions [27,28,29]. Our earlier work established the waste film apparatus and examined pulsed PE cleaning and a water-washing reference [30]. Its optical and first gravimetric recovery expressions are algebraically equivalent to the present optical and unrestricted apparent mass indices. The authors identify the retained records as a separate experimental series collected with this apparatus and these endpoint definitions. Earlier run-level records and a complete acquisition chronology were unavailable for direct record-to-record comparison, so dataset independence rests on author attestation. Tables S1 and S19 distinguish the reused apparatus and methods, current records, derived analyses and separate measurement datasets.

The present study asks how incomplete recovery changes the observations that support a cleaning interpretation. It quantifies the coverage and selection imposed by a count-matched weighing criterion, estimates the scale of missing film contributions relative to apparent contamination increments, and examines optical sampling variability and handling blanks. Each analysis links a comparison to the film population sampled or weighed, allowing for the applicability of mass-based and optical measurements to be assessed across the tested operating conditions.

## 2. Materials and Methods

### 2.1. Study Design and Data Structure

**Figure 1:**
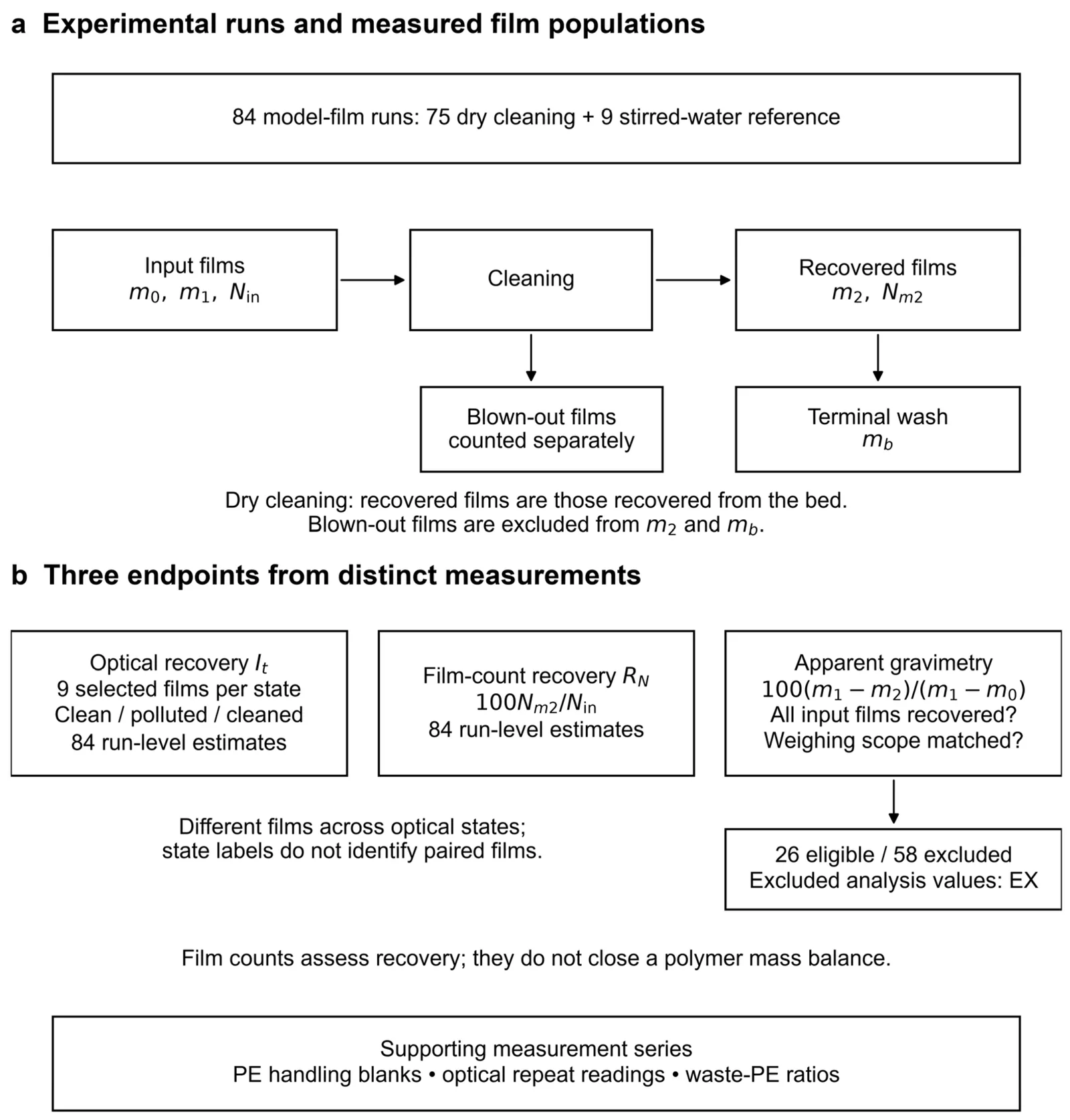
Study design and measurement populations. The 84 model film runs comprise 75 dry cleaning runs and nine laboratory stirred water reference runs. Optical measurements comprise nine separately selected films per state, without tracking individual films across states; the selection and drying procedures are retrospectively reported by the authors (Section 2.4). For dry cleaning runs, the reported post-cleaning optical sample was drawn from bed-recovered films. For dry cleaning, *m*_0_ and *m*_1_ refer to all input films, whereas *m*_2_ and *m_b_* refer to the bed-recovered films included in weighing. Blown-out pieces were counted separately and were not included in *m*_2_. The count-matched, consistent scope rule retains 26 apparent gravimetric observations and marks 58 as excluded (EX) for that endpoint. Film count recovery uses the weighed-to-input count ratio and is not a polymer mass yield. Waste polyethylene (PE) ratios and separate repeat reading data do not add runs to the 84-run analysis.

The model film dataset comprised 84 runs with unique Run_IDs: 75 dry cleaning runs and nine laboratory stirred water washing reference runs (Figure 1; Table 1). The materials were PE, PP, polyethylene terephthalate (PET) and an equal-mass mixture of these polymers (MIX). Comparisons covered material–geometry configurations, duration-matched protocols, nominal humidity settings and film loading; a further 13 records carry sand history source labels. VAL-COMP supplied the shared PE reference and one model confirmation group; its three runs were counted once in the dataset. Terminal wash ratios from a separate study of 12 waste PE source batches are summarized in Section S9. These ratios were analyzed separately from the model-film dataset.

The dataset contains masses, film counts, operating settings and 2268 single-film optical readings. Optical recovery was calculated from the three state means, film count recovery from the recovered-to-input count ratio, and gravimetric eligibility from count matching and consistent weighing scope. Supplementary_Data.xlsx provides the measurements and calculated endpoints. The retained source tables permit recalculation but are not instrument-native acquisition files. Complete acquisition dates, preparation batch histories and some instrument records were unavailable. Table S19 distinguishes retained records, derived quantities, retrospective author confirmations and evidence that cannot be independently verified. Numerical reproducibility does not establish verification of the acquisition history. Procedure details supported by retrospective author confirmation are identified below, separately from contemporaneous records.

### 2.2. Model Films, Contamination Preparation and Pulsed Cleaning

Commercial transparent PE, PP and PET films were purchased through an online marketplace. The authors report center-point thickness values of 30, 32 and 25 μm, respectively, obtained with a GREENER digital outside micrometer (0–25 mm range; 0.001 mm display resolution). The PE and PP values are attributed to remeasurement of original lot material by retrospective author confirmation; the PET measurement cannot be linked to the original experimental lot. Individual thickness readings, specimen identifiers and measurement dates were not retained. The PET value is therefore a reported material descriptor, not a verified thickness of the tested lot. Polymer grade, molecular weight distribution, film-forming method and additive composition were unavailable. The comparisons concern the tested commercial material–geometry configurations, not isolated effects of polymer chemistry or matched thickness. Table S20 summarizes the material and instrument information and its evidential basis.

Films were cut into approximately square pieces with a nominal cutting target of 30 ± 2 mm per side; this value is not a measured mean and standard deviation of film dimensions. Standard loadings were approximately 3 g of clean film: 120 PE pieces, 115–116 PP pieces, 97 PET pieces or 107–108 MIX pieces. MIX contained equal masses of PE, PP and PET, rather than equal film areas. The higher PE loading was approximately 5 g and 200 pieces. Run masses and counts are given in S1a_Cleaning and operating conditions in S1b_Conditions. Contamination loading was specified by input mass ratios rather than individual film area.

Contamination preparation used soil collected on the Tongji University campus and dried at 60 °C for 48 h. For soil contamination, film, dried soil and purified water were mixed at film-to-soil and film-to-water mass ratios of 1:3 and 1:10, respectively, followed by drying at 50 °C for 24 h. For oil–dust contamination, films were first mixed with edible oil at a film-to-oil mass ratio of 1:0.25; dried soil was then added at a film-to-soil mass ratio of 1:10, followed by drying at 50 °C for 6 h. These are preparation charge ratios. The apparent contamination increment used in the gravimetric calculation was the difference between the contaminated and clean masses of the same input film set.

The cylindrical cleaning section had a 200 mm internal diameter and a height of 650 mm. A 240 mm expansion section above it widened to a top internal diameter of 300 mm. A lateral outlet connected the bed to a particle collection unit. The distributor contained 57 caps, each with four 3 mm holes. The medium was 40–70 mesh quartz sand with a nominal initial loading of 2 kg. A central control box varied blower output through a variable-frequency drive in cyclic simple programmable logic controller (PLC) operation. Selection of parameters through the integrated controller’s built-in program is described by retrospective author confirmation. Complete manufacturer/model identifiers for the controller, drive and blower were not retained (Table S20).

The common superficial velocity, amplitude and frequency settings were 0.68 m·s^−1^, 0.12 m·s^−1^ and 1.50 Hz. These values describe controller setpoints; mean flow measurements do not resolve the instantaneous waveform or the films’ hydrodynamic exposure. Settings for the 17-run Box–Behnken design (BBD) and nine confirmation runs are provided in Section S3. The control target implementation in Supporting_Files.zip illustrates the sinusoidal setpoints mathematically; it is not the controller’s original program or a measurement of delivered airflow.

Timing began when contaminated films entered the sand bed. After gas flow stopped, bed-retained films, sand and material in the collection unit were recovered separately. Input, bed-recovered and separately collected outlet pieces were recorded separately; only bed-recovered films contributed to *m*_2_ in dry cleaning runs. The retained count totals do not provide fragment-level inspection or duplicate counting records. Agreement of whole-piece counts therefore does not exclude unobserved fines, partial film loss or counting uncertainty. Films were dried at 45–50 °C for 24 h before weighing.

### 2.3. Laboratory Stirred Water Reference and Operating-Condition Experiments

The 600 s observations used soil-contaminated PE, PP and PET, with three runs per material and protocol. Earlier equipment studies also compared dry cleaning with water washing [31]. Here, the laboratory stirred water reference used approximately 3 g of clean film input in a 100 mL glass beaker containing 50 mL of fresh deionized water for each run. Films were stirred at 50 rpm for 600 s without an additional rinse. Post-treatment drying was 45–50 °C for 24 h before weighing. This reference was distinct from terminal ultrasonic washing (Section 2.4). Equal duration did not match energy input, mixing intensity, medium, handling or the complete drying chain between protocols.

Separate 120 s experiments compared the four material–geometry configurations and, for PE, nominal relative humidity (RH) settings of 60% and 80% and nominal film loadings of 3 and 5 g. Each group comprised three recorded runs. The reported environmental adjustment protocol used air conditioning, an evaporative humidifier, a dehumidifier and circulation fans, with readings at the blower intake and bed inlet to adjust the intended inlet RH. Sand and film moisture were not independently measured or controlled, so the groups are identified by nominal RH setting. Retrospective author confirmation identifies film mass and count as the only intended changes in preparation or operating conditions for the loading comparison. Film-to-sand ratios use the recorded clean film and sand masses.

Thirteen 600 s records for oil–dust-contaminated PE carry the source labels BOUND-OIL-CYCLE (ten records) and BOUND-OIL-RESTORE (three records). The source notes use repeated use and post-heating labels; retrospective author confirmation specifies a 600 °C, 5 h heat treatment. The recorded sand masses could not be reconciled with a verified run-to-run transfer history (Section S3; Table S19). These labels are retained for record identification, not treated as a validated continuous sand batch experiment. The film endpoints are reported descriptively, without inference about cumulative use, thermal recovery or regeneration.

### 2.4. Optical, Count and Apparent Gravimetric Measurements

Visible-light transmittance was measured with the newer LS116 meter (Shenzhen Linshang Technology Co., Ltd., Shenzhen, China), with a display increment of 0.01 percentage point over the measured range. For each run, one central reading was taken on each of nine films in each of the clean, contaminated and post-cleaning states. According to retrospective author confirmation, films were mixed and nine films were selected without replacement, without excluding difficult-to-measure films. Individual films were not tracked across states, and the post-cleaning dry treatment sample came from bed-recovered films. No contemporaneous sampling list or recorded randomization procedure was retained, so selection probabilities and selection bias cannot be independently reconstructed. The authors retrospectively identify all three optical states as measured after the stated drying procedures. State-specific cooling times and equilibration logs were not retained; equivalent moisture equilibration across states is not independently verified. Optical summaries and conditional sampling uncertainty refer to the selected films.

The nine readings were averaged within each state to obtain, ${\bar{T}}_{0}$, ${\bar{T}}_{1}$ and ${\bar{T}}_{2}$, and optical recovery was calculated from those means:

(1) $$I_{t}=100\frac{{\bar{T}}_{2}-{\bar{T}}_{1}}{{\bar{T}}_{0}-{\bar{T}}_{1}}$$

*I_t_* = 0% denotes equality of the post-cleaning and contaminated state means; *I_t_* = 100% denotes equality with the sampled clean reference mean. Values above 100% would indicate a higher post-cleaning mean than the sampled clean reference and could arise from sampling, optical changes or measurement variation. They would not represent removal of more than 100% of contamination. The index is not truncated at 0% or 100%. The 2268 readings, state summaries and denominator values are provided in Section S6 and Table S23.

Film count recovery used the number of films included in post-cleaning weighing, *N_m_*_2_, relative to the input count, *N*_in_:

(2) $$R_{N}=100\frac{N_{m2}}{N_{\mathrm{in}}}$$

For dry cleaning, *R_N_* describes pieces recovered from the bed; for the stirred water reference, it describes pieces recovered for weighing after stirring. Blown-out pieces do not enter the dry cleaning numerator. *R_N_* is a recovery count at the specified collection location and does not measure polymer mass yield.

Masses were measured with an AL204-IC analytical balance (METTLER TOLEDO; readability 0.1 mg). Retrospective author confirmation describes individual stable net readings after taring. Calibration certificates and contemporaneous check-mass results were unavailable, and readability was not treated as measurement uncertainty. The masses *m*_0_ and *m*_1_ refer to the clean and contaminated full input film set, while *m*_2_ refers to the recovered set entering post-cleaning weighing; the population correspondence is supported by the retained fields and retrospective author confirmation, not specimen-level weighing lists. Apparent gravimetric change was calculated as

(3) $$I_{m,\mathrm{app}}=100\frac{m_{1}-m_{2}}{m_{1}-m_{0}}$$

A gravimetric value was included when mass and count data were available, *N_m_*_2_ = *N*_in_ and the weighing scope was consistent. The corresponding calculation without this restriction is reported separately as *I*_unrestricted_. EX denotes exclusion from the apparent gravimetric endpoint, not zero mass change or an invalid optical observation. Matching whole-piece counts does not exclude abrasion, leaching or cleaning medium retention.

Terminal ultrasonic washing used the same recovered films contributing to *m*_2_. A 100 mL glass beaker containing 60 mL of deionized water was placed in a KQ3200DE cleaner (Shumei, Kunshan Ultrasonic Instruments Co., Ltd., Kunshan, China), operated at 40 kHz and the 100% power setting for two 180 s cycles. The nameplate ultrasonic input power was 150 W. Water was replaced between cycles; the heater was off, initial water was at room temperature, and no additional rinse was used. Terminal drying was performed at 60 °C, and the final mass was designated *m_b_*. Thus, *m*_2_ and *m_b_* describe the same recovered-film set following different drying procedures. Section S10 gives the terminal washing protocol.

The operational terminal wash mass change ratio, *R_d_*, and apparent mass deficit, *L*_app_, were

(4) $$R_{d}=100\frac{m_{2}-m_{b}}{m_{2}}$$

(5) $$L_{\mathrm{app}}=100(1-\frac{m_{b}}{m_{0}})$$

*R_d_* compares masses of the same recovered films before and after terminal washing. *L*_app_ additionally requires the count-matched weighing scope because it compares *m_b_* with the full-input mass *m*_0_. These quantities combine operational effects of washing, drying and handling; they do not partition contaminant removal from polymer change. Their definitions, eligibility and values are provided in Sections S10 and S12 and Figure S1.

A separate optical repeat-reading dataset comprised 27 films, with three films for each PE, PP and PET material–state combination. Each film was read ten times at the same position and ten times after removal and repositioning, yielding 270 readings per mode. The retained CSVs contain no acquisition timestamps or links between validation specimens and cleaning Run_IDs. These measurements characterize within-film reading and repositioning variability; they do not establish contemporaneous validation of the 84-run campaign or increase its number of cleaning runs.

The PE blank dataset comprised 12 records across four conditions: drying-only, terminal wash-only, 120 s gas–solid handling followed by terminal washing, and 600 s gas–solid handling followed by terminal washing, with three records per condition. The retained blank records do not identify acquisition dates, material batch identifiers or matched cleaning Run_IDs. They are used as a separate, stage-specific PE sensitivity dataset. Correspondence to the contaminated film experiments in time and material batch is not established. Mass changes were calculated separately for the pre-terminal, terminal wash and complete sequences (Section S11; Table S25). The blank measurements include 60 °C drying readings after 4, 5 and 6 h. Each stage calculation uses its corresponding weighing values. These blanks were analyzed separately from the 84 model film observations.

### 2.5. Illustrative SEM–EDS Observations

PE surfaces were examined with a Sigma 300 scanning electron microscope (SEM; ZEISS) and SE2 detector at 15 kV and 1000× magnification, using gold coating and mechanical clamping. PE1, PE2, PE3 and PE4 identify clean, soil-contaminated, stirred water reference and dry-cleaned states. One field per state is analyzed; the images are not linked to individual cleaning Run_IDs. Working distances were 7.7, 7.7, 7.9 and 7.8 mm, respectively. The full fields and scale bars are presented with the surface observations in Section 3.4.

Energy-dispersive X-ray spectroscopy (EDS) measurements were performed by an external testing organization. The reports list C, O, Na, Mg, Al, Si, K and Ca, with mass fractions normalized to 100% within this set. Au is absent from the listed elements, although the samples were gold-coated. The quantification method and coating correction were unspecified, so the fractions are used as local illustrative observations rather than quantitative route comparisons (Tables S5–S8). Reported sigma values describe counting or fitting uncertainty, not between-specimen standard deviations.

### 2.6. Statistical Analysis

The cleaning run was the analysis unit. Within-condition summaries give individual observations, means and sample standard deviations (SDs), with each endpoint’s valid *n*. For *n* ≥ 2, exploratory conditional 95% mean intervals were calculated as mean ± *t*_0.975,*n*−1_ SD/$\sqrt{n}$ [32]. Single eligible values have no SD or interval; groups with no eligible gravimetric value are marked EX. Identical observed counts may yield SD = 0, which does not imply zero population uncertainty; the count step is at least 100/*N*_in_ percentage points. No replicate-based interval was assigned to the mixed BBD design or the 13 source-labelled sand history records. Retrospective author confirmation identifies the three VAL-COMP observations at 120 s as separately prepared film groups, each loaded, treated and recovered individually. This account does not establish independent preparation batches or remove possible dependence associated with shared sand or run order.

Five specified optical contrasts used unpaired Welch 95% intervals [33]: stirred water minus dry cleaning for PE, PP and PET at 600 s, nominal 80% minus 60% RH, and nominal 5 g minus 3 g PE loading. Each comparison used the three runs in each group, with a Welch–Satterthwaite degrees-of-freedom calculation. The intervals are conditional on independent runs and approximately normal run-level variation; *n* = 3 provides little ability to assess those assumptions. They were not adjusted for multiple comparisons and were not interpreted as causal, equivalence or industrial superiority tests. Formulae are given in Section S12; endpoint-specific group intervals are provided in R1_Group_CI, and the five optical contrasts are summarized in Table S24 and R1_Contrasts.

Optical sampling sensitivity used the sample variance of the nine readings in each state. With *D* = ${\bar{T}}_{0}$ − ${\bar{T}}_{1}$, the derivatives of *I_t_* are *g*_0_ = −*I_t_*/*D*, *g*_1_ = (*I_t_* − 100)/*D* and *g*_2_ = 100/*D*. First-order propagation [34], conditional on independent state means, gives

(6) $${u_{\mathrm{sampling}}}^{2}(I_{t})={g_{0}}^{2}\frac{{s_{0}}^{2}}{9}+{g_{1}}^{2}\frac{{s_{1}}^{2}}{9}+{g_{2}}^{2}\frac{{s_{2}}^{2}}{9}$$

The corresponding denominator component is *u*_sampling_(*D*) = $\sqrt{s_{0}^{2}/9+s_{1}^{2}/9}$. These estimates quantify variability within the selected film samples and exclude systematic instrument effects, shared drift, selection effects and batch correlation. A supplementary conditional bootstrap sampled nine readings with replacement separately within each state for 10,000 replicates, using seed 20,261,008; the 2.5th and 97.5th percentiles define the reported interval [35]. No cross-state film pairing was imposed. Display rounding sensitivity was assessed by perturbing each state mean by ±0.005 percentage point at all eight corners. This half-display-step perturbation is a rounding sensitivity analysis, not an accuracy specification or confidence limit. Table S23 distinguishes these quantities.

For incomplete recovery runs, missing film scenarios set *q* = *α*(*N*_in_ − *N_m_*_2_)*m*_0_/*N*_in_, with illustrative *α* values of 0.5, 1.0 and 1.5. The shift Δ*I* = 100*q*/(*m*_1_ − *m*_0_) was subtracted from *I*_unrestricted_ to display the possible scale of unweighed clean film mass. Neither *q* nor the resulting scenario index was treated as a measurement or used to restore eligibility. The actual missing pieces could differ in mass and retain contamination. A separate PE blank-scale analysis used 100|*b*_pre_|/(*m*_1_ − *m*_0_), where *b*_pre_ is the pre-terminal blank mass change, for eligible PE 120 s runs. All scenario fields remain separate from observed masses and endpoint values (Tables S22 and S25).

Linear, additive quadratic and full quadratic optical models were fitted to the same 17 Box–Behnken observations, with 4, 7 and 10 coefficients. The nine confirmation runs were excluded from fitting. Section S13 distinguishes fitted mean confidence intervals, single-run prediction intervals and prediction intervals for a future group mean, and reports heteroscedasticity-robust (HC3) standard errors, leave-one-out error, residual structure and omission sensitivity. These analyses assess model behavior within the experimental design; they do not test prediction across new preparation batches or dates.

Calculations used Python 3.12.10, NumPy 2.4.6 and SciPy 1.18.0; figures were prepared with Matplotlib 3.11.1. Supporting_Files.zip provides the analysis and plotting scripts, required inputs, software versions and reproduction commands.

## 3. Results

### 3.1. Gravimetric Coverage and Missing-Film Magnitude

Optical recovery and film count recovery were calculable for all 84 model film runs, whereas 26 runs (31.0%) met the apparent gravimetric inclusion rule. Eligibility differed by protocol: 18 of 75 dry cleaning runs (24.0%) and eight of nine stirred water reference runs (88.9%) had count-matched weighing populations. Ten of the 21 groups had no eligible gravimetric observation and three had one (Table S21). In the 17-run BBD design, six values were eligible, fewer than the ten coefficients in a full quadratic gravimetric model. The remaining 58 runs retain their optical and count observations and their separately labeled unrestricted mass calculations.

The inclusion rule selects complete recovered film sets directly through *R_N_*. The 26-run gravimetric subset therefore cannot represent all tested conditions or all dry cleaning runs. Within-group eligible and excluded observations are reported in Table S21; similarity between pooled optical means, if present, would not remove this deterministic selection. Groups with no eligible mass observation cannot support a joint three-endpoint comparison.

BBD-R02 contained 120 input pieces but 119 pieces in the post-cleaning weighing, with one reported blown-out piece. Its *m*_0_, *m*_1_ and *m*_2_ values were 3.0017, 3.1301 and 3.0032 g. The apparent contamination increment was 128.4 mg, and direct substitution gave *I*_unrestricted_ = 98.8%. The mean clean film mass was 25.0 mg per input piece. Assigning that mean mass to the unweighed piece gives a scenario contribution of 19.5 percentage points to the unrestricted index. The actual mass and residual contamination of that piece were not measured, so this calculation identifies a potential bias magnitude without correcting the observation. Table S22 shows three BBD-R02 scenarios; complete run–scenario records are provided in Supplementary_Data.xlsx.

### 3.2. Material–Geometry Configurations and Duration-Matched Laboratory Observations

#### 3.2.1. Separate 120 s Material Observations

**Figure 2:**
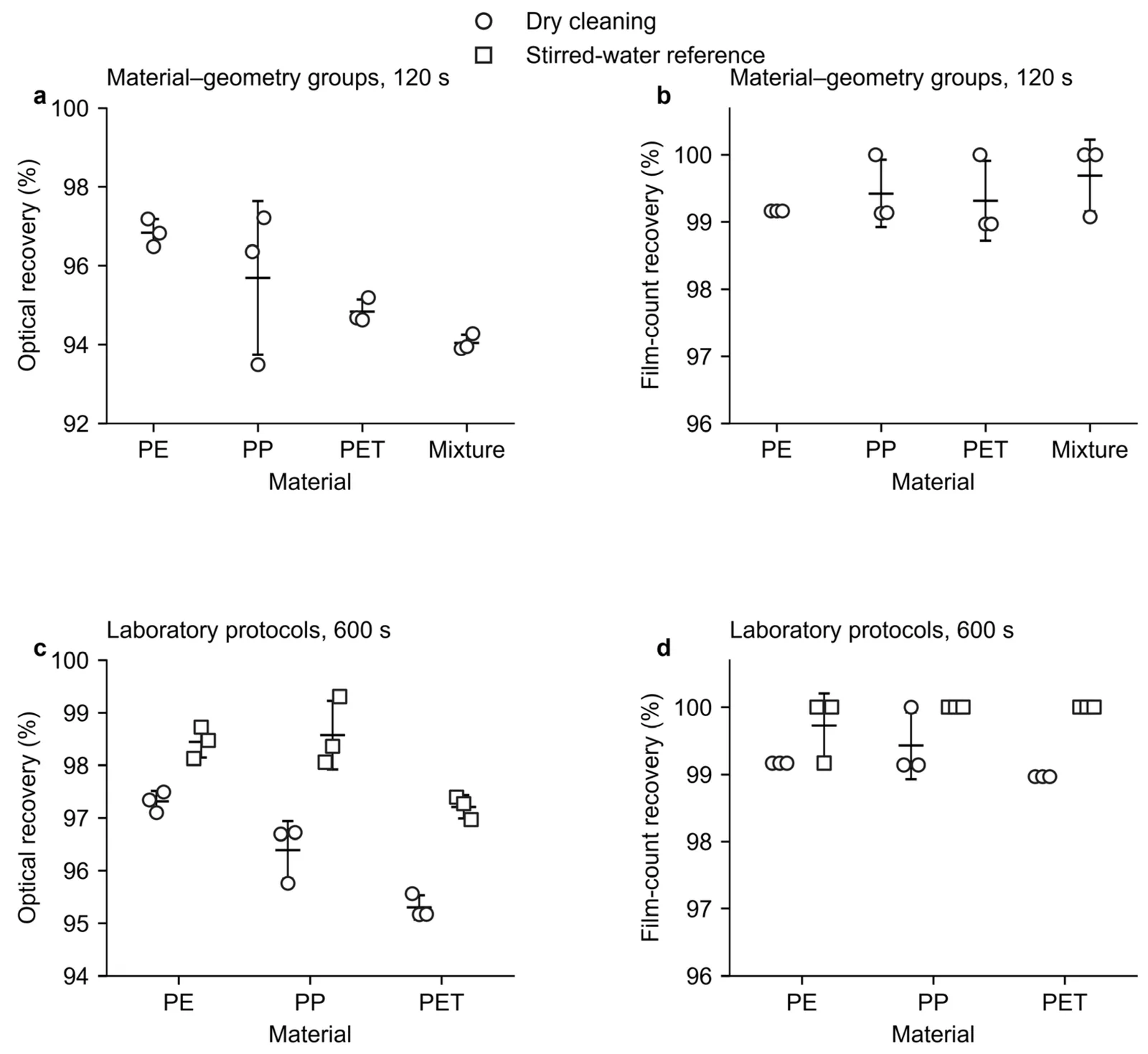
Optical recovery, *I_t_*, and film count recovery, *R_N_*, in separate 120 s and 600 s experiments. (**a**,**b**) Optical and count observations for PE, PP, PET and their equal-mass mixture (MIX) at 120 s. (**c**,**d**) Optical and count observations for the dry cleaning and laboratory stirred water reference protocols at 600 s. PE, PP and PET denote polyethylene, polypropylene and polyethylene terephthalate, respectively. Points denote recorded runs; horizontal bars and error bars show means ± sample standard deviation (SD; three runs per group). Different endpoints for a run do not add replication. According to retrospective author confirmation, the 120 s and 600 s experiments used different films from the same procurement/preparation batch; they do not form a treatment time trajectory. This statement does not establish batch independence. Conditional mean and contrast intervals are reported in Table 2 and Table S24. The VAL-COMP PE reference is reused where indicated and counted once among the 84 runs.

After 120 s under the common pulsed setting, mean *I_t_* was 96.8 ± 0.3% for PE, 95.7 ± 1.9% for PP, 94.8 ± 0.3% for PET and 94.0 ± 0.2% for MIX (*n* = 3 per group; Figure 2a). The corresponding mean *R_N_* values were 99.2%, 99.4%, 99.3% and 99.7% (Figure 2b). These observations compare commercial material–geometry configurations with different reported thicknesses and piece counts; the PET thickness is not linked to the tested lot. They do not isolate polymer identity as the cause of the optical ordering.

#### 3.2.2. Duration-Matched Laboratory Observations

At 600 s, the observed stirred water-minus-dry optical differences were 1.1, 2.2 and 1.9 percentage points for PE, PP and PET, respectively. The corresponding exploratory conditional 95% intervals were 0.5–1.7, 0.8–3.6 and 1.4–2.4 percentage points (Table S24). Table 2 reports the endpoint-specific observations, and Figure 2c,d shows the individual runs. Equal treatment duration left energy input, mixing, medium and the full handling and drying chains unmatched. The contrasts therefore describe the specified laboratory protocols. No dry cleaning PE or PET run was eligible for apparent gravimetric comparison; the dry cleaning PP group contained one eligible value.

Mean *I_t_* in the constant flow groups ranged from 69.4% to 78.0%, compared with 96.8% for the shared pulsed setting reference (Figure S2). These observations describe controller setting groups; setpoints and mean flow measurements do not resolve instantaneous flow. The descriptive optical model results are provided in Section S13.

### 3.3. Nominal RH Setting, Film Loading and Source-Labelled Sand Records

**Figure 3:**
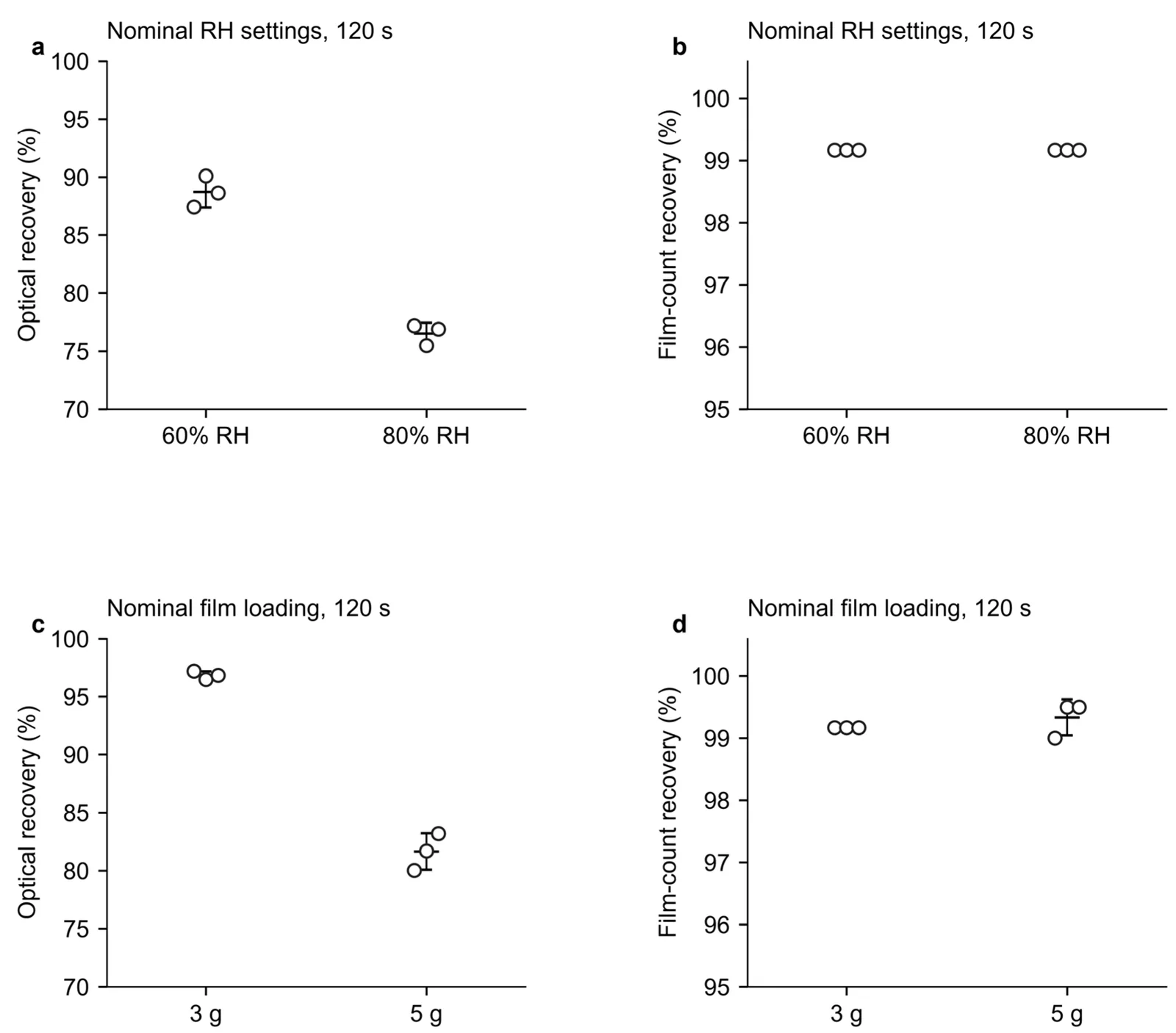
Optical recovery, *I_t_*, and film count recovery, *R_N_*, at nominal RH settings and PE loadings at 120 s. (**a**,**b**) Nominal 60% and 80% RH settings. (**c**,**d**) Nominal 3 g and 5 g PE loadings, with 120 and 200 input pieces per run, respectively. Open circles show individual runs; horizontal bars and vertical error bars indicate group means and ±1 sample standard deviation, respectively (*n* = 3 per group). The RH labels describe environmental settings, not measured film or sand moisture. The loading comparison changes film mass and piece count together. Conditional contrast intervals are in Table S24. VAL-COMP is the shared 3 g reference.

At the nominal 60% and 80% RH settings, mean *I_t_* was 88.7 ± 1.3% and 76.5 ± 0.9%, respectively; both groups had mean *R_N_* = 99.2% (Figure 3a,b). The 80% minus 60% RH optical difference was −12.2 percentage points, with an exploratory conditional 95% interval of −14.9 to −9.5 percentage points (Table S24). These differences describe the nominal environmental settings; they do not identify the moisture exposure or adhesion mechanism at the film and sand surfaces.

The nominal 5 g PE group contained 4.9903–5.0038 g and 200 input pieces per run; the nominal 3 g reference contained 2.9934–3.0044 g and 120 pieces per run. Recorded film-to-sand ratios were 2.494–2.503 g·kg^−1^ and 1.499–1.502 g·kg^−1^, respectively. Mean *I_t_* was 81.6 ± 1.6% at 5 g and 96.8 ± 0.3% at 3 g. The difference, 5 g minus 3 g, was −15.2 percentage points (conditional 95% interval, −18.9 to −11.5), while mean *R_N_* remained close to 99% (Figure 3c,d). Film mass, piece count and bed occupancy changed together, so the observations do not isolate film–film interactions, shielding or film–sand contact, or define an industrial throughput limit.

**Figure 4:**
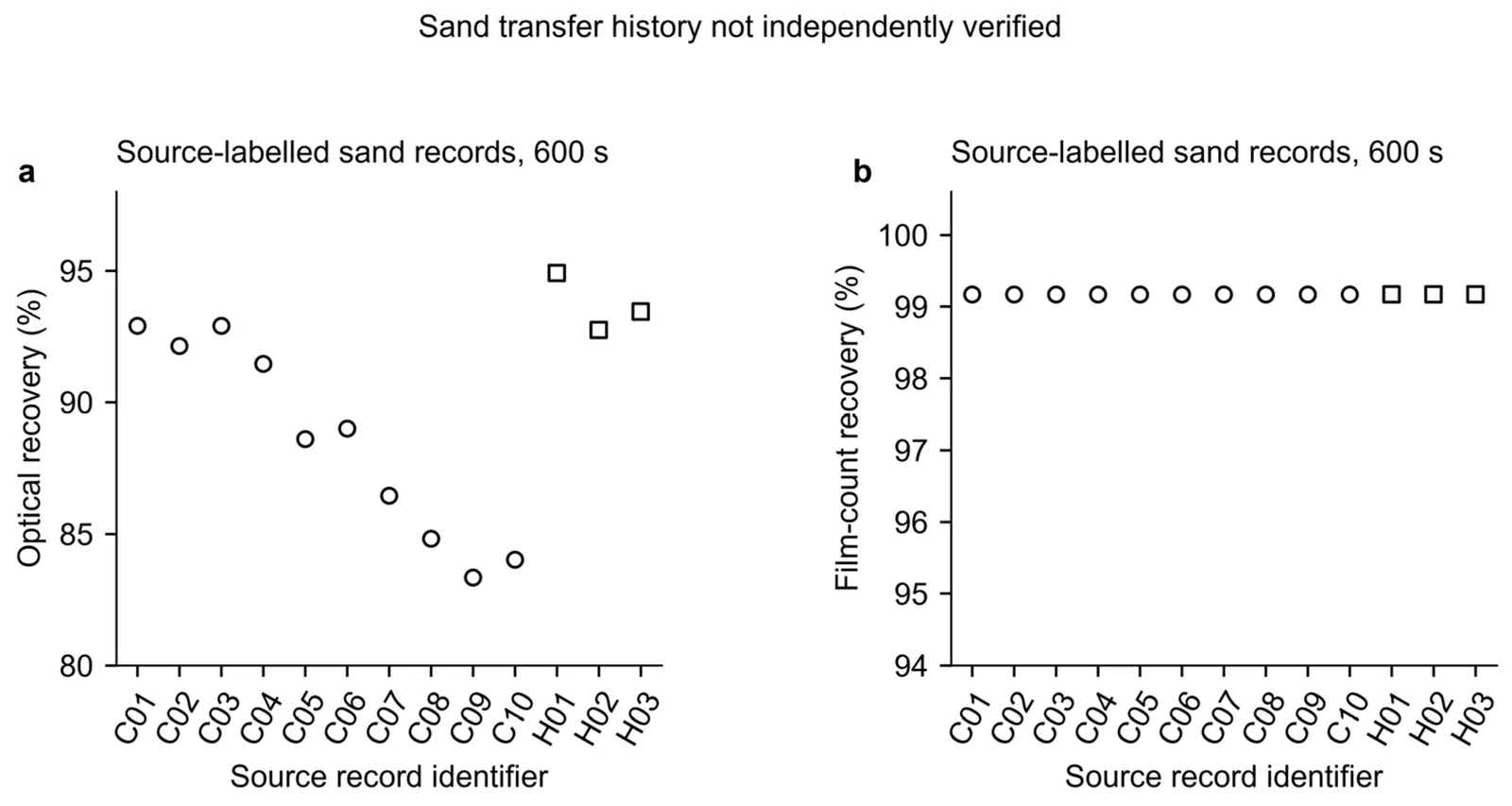
Optical and film count endpoints for the 13 records labelled BOUND-OIL-CYCLE and BOUND-OIL-RESTORE, each at 600 s. (**a**) Optical recovery, *I_t_*. (**b**) Film count recovery, *R_N_*. Each unconnected symbol represents one recorded cleaning run. C01–C10 and H01–H03 retain source record identifiers, not an independently verified acquisition chronology. Sand transfer history is not verified: the sand mass and transfer records do not establish batch continuity. These points are not used to infer a cumulative use trajectory, heating effect or regeneration performance, and no replicate-style means, error bars or heating comparisons are assigned. Open circles denote BOUND-OIL-CYCLE records (C01–C10), and open squares denote BOUND-OIL-RESTORE records (H01–H03).

Among the BOUND-OIL-CYCLE records, *I_t_* was 92.9% for C01 and 84.0% for C10 (Figure 4a). The BOUND-OIL-RESTORE records H01, H02 and H03 had *I_t_* values of 94.9%, 92.8% and 93.5%, respectively. *R_N_* was 99.2% for all 13 records (Figure 4b). These are record-level descriptions: the sand mass and transfer information does not establish batch continuity or an acquisition chronology. The endpoints therefore do not define a cumulative use trajectory, a heating effect, regeneration performance or a service life interval.

### 3.4. Illustrative Local PE Surface Observations

**Figure 5:**
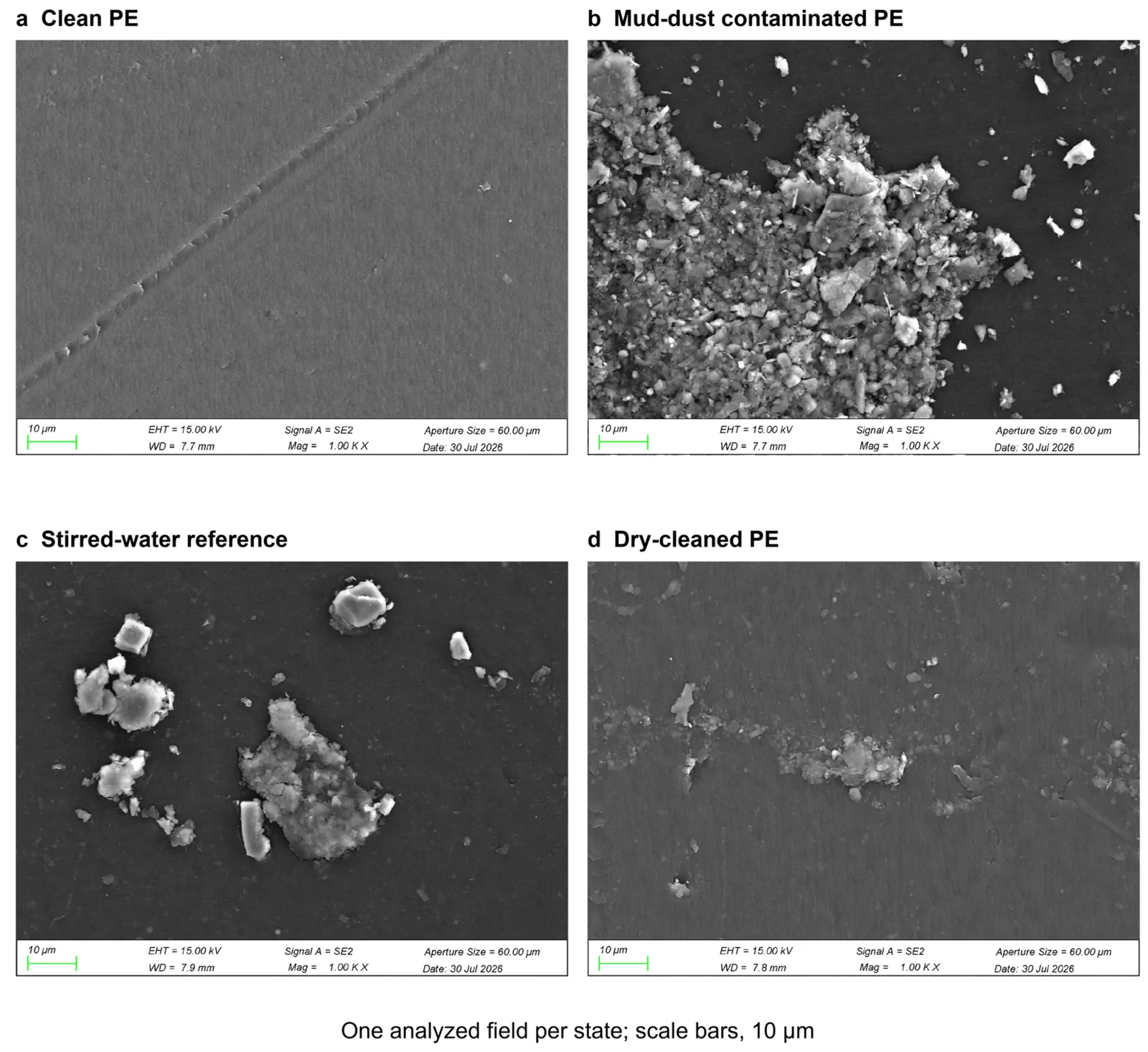
Local PE surface fields: (**a**) clean PE (PE1), (**b**) soil-contaminated PE (PE2), (**c**) stirred water reference PE (PE3) and (**d**) dry-cleaned PE (PE4). The full fields and 10 μm scale bars are shown. Acquisition conditions were 15 kV and 1000× magnification, with working distances of 7.7, 7.7, 7.9 and 7.8 mm for PE1–PE4. Samples were gold-coated and mechanically clamped. One field per state is analyzed for local morphological illustration. Energy-dispersive X-ray spectroscopy (EDS) fractions, normalization and quantification details are given in Tables S5–S8; report sigma values are not independent-specimen SDs.

The clean PE field showed continuous film texture, whereas the soil-contaminated field contained dense particles and larger agglomerates. Dispersed particles or localized agglomerates remained in both cleaned fields (Figure 5). These images illustrate local surface morphology and residual material. The elemental outputs are provided in Tables S5–S8 as descriptions of the analyzed fields, without a quantitative ranking of cleaning routes.

### 3.5. Optical Sampling Variability and Stage-Specific PE Blank Responses

The 252 state summaries showed between-selected-film transmittance SDs of 0.45–1.79 percentage points in the clean state, 0.46–1.66 in the contaminated state and 0.23–1.59 after cleaning. The normalization denominator *D* ranged from 68.0 to 76.2 percentage points. Conditional propagation of the state variances gave *u*_sampling_(*I_t_*) = 0.33–0.99 percentage point, and the minimum *D*/*u*_sampling_(*D*) was approximately 98.8 (Table S23). The present denominators were therefore well separated from zero relative to this sampling component. This result concerns the tested contamination range; it does not validate the index for lightly contaminated films or account for common systematic effects. The maximum change in the display-rounding check was approximately 0.0147 percentage point.

In the separate optical repeat reading dataset, the mean within-film same-position SD across the nine material–state combinations ranged from 0.022 to 0.041 percentage point, and the corresponding repositioning SD ranged from 0.165 to 0.237. These values refer to transmittance readings, not directly to uncertainty in *I_t_*. They characterize variation on repeatedly measured films and do not replace the between-film state variances used above or establish contemporaneous validation of the cleaning campaign. Repositioning variance was not added again to the observed state variances, which already contain reading variation.

The PE 120 s handling blanks had pre-terminal mass changes of −1.7 to −1.1 mg, with a mean of −1.4 mg. Relative to the apparent contamination increments of the 13 eligible PE 120 s runs, this range corresponds to a gravimetric index scenario magnitude of 0.82–1.49 percentage points (Table S25). The 600 s pre-terminal blank changes were −2.0 to −1.5 mg. Mean total changes after terminal washing were approximately −4.1 mg for the 120 s blanks and −5.4 mg for the 600 s blanks; those totals were not used to correct the pre-terminal *m*_2_ endpoint.

PE blanks were used to assess the scale of handling-related mass changes without subtracting them from the cleaning endpoints. Their response has not been shown to transfer quantitatively to contaminated films, and they do not quantify abrasion or sand retention in those films. The blank analysis applies to PE; it does not provide corrections for PP, PET or MIX. The PE 600 s dry cleaning group has no eligible apparent gravimetric value, so its blank results cannot create a corrected group mean. Recovery of all 120 pieces in the blanks coexisted with nonzero mass changes.

The optical models remained descriptive. The additive and full quadratic models had adjusted *R*^2^ values of 0.9169 and 0.8949 and leave-one-out errors of 0.886 and 0.787 percentage point, respectively. The full quadratic VAL-LE confirmation mean lay below its conditional prediction interval for a mean of three runs. Section S13 provides all three model specifications and separates this group mean interval from the wider single-run prediction interval.

## 4. Discussion

### 4.1. Weighing Populations Determine Gravimetric Coverage

The central measurement result is the uneven coverage imposed by complete film recovery. A rule that retains 24.0% of dry cleaning runs and 88.9% of stirred water reference runs changes which protocols and conditions can be compared by mass. It selects directly on recovered piece completeness; an eligible subset cannot be treated as an unbiased summary of the original experimental population. Optical and count observations remain informative for excluded runs, but they do not supply the missing gravimetric observations. Reporting endpoint availability by group makes that distinction visible before interpreting response magnitudes.

The BBD-R02 scenario explains why this restriction matters even when an unrestricted index is near 100%. An average clean piece represents 19.5 percentage points of that run’s apparent contamination increment. Its omission can therefore contribute appreciably to the numerator without being contaminant removal. Actual missing piece mass and retained contamination remain unknown, and matching whole-piece counts would still leave abrasion and medium retention unresolved. Related material flow and odor studies distinguish recovery quantity from product quality attributes [36,37]; in the present experiments, the additional distinction is the measured film population attached to each endpoint.

### 4.2. Finite Configuration Contrasts and Unidentified Mechanisms

The 120 s loading contrast is informative because optical recovery changed by about 15 percentage points while film count recovery stayed near 99%. Piece recovery alone therefore cannot predict the optical condition of selected recovered films. The comparison jointly changes mass, piece count and bed occupancy, however, and provides no separate measurement of shielding, contact frequency or film motion. Likewise, the 600 s optical contrasts quantify differences between specified laboratory protocols; their conditional intervals do not match the protocols’ energy, mixing or downstream processing requirements.

Nominal RH settings do not specify film and sand moisture, so the present observations cannot explain the different qualitative humidity response in the earlier study [30]. Humidity-dependent adhesion has been examined for other surfaces and controlled geometries [38,39,40,41]. Those studies identify variables relevant to cleaning but do not establish the mechanism in the present experiments. The 13 source-labelled sand records are descriptive only: their transfer history cannot be reconstructed sufficiently to interpret them as consecutive uses of one batch or as a heating comparison. Regeneration repeatability would require a documented transfer history and independent sand batch comparisons.

### 4.3. Sampling, Blanks and Material Information

The optical analysis separates three sources of numerical variation that serve different purposes: differences among selected films, repeated readings on a film, and display rounding. Between-film state variances enter the propagated sampling component; the separate same-position and repositioning measurements characterize the instrument–film measurement procedure. The current denominator range is large relative to its estimated sampling component. When the initial transmittance loss is small, the denominator can become comparable to its uncertainty, and a stable normalized result cannot be assumed. Reporting all three state means and obtaining independent reference measurements would then be necessary.

Stage alignment determines which blank change is relevant to an endpoint. For the eligible PE 120 s runs, the retained pre-terminal blank range gives a sensitivity scale of approximately one percentage point in the apparent gravimetric index. This calculation does not establish run-level or batch-level matching. A correction would additionally require justified transfer of the blank response to contaminated specimens and propagation of its uncertainty. Total changes after terminal washing belong to a later stage and are not used to correct the pre-terminal endpoint. Comparisons among the commercial films also combine polymer identity with geometry and material structure. Without material-specific molecular weight and composition data, and with the PET thickness unlinked to the tested lot, the observed ordering cannot be attributed to polymer chemistry alone.

### 4.4. Model Scope and Component-Resolved Mass Balance

The optical models characterize responses within the tested design, with confirmation runs evaluated separately from model fitting. Leave-one-out analysis measures sensitivity to observations within that design. It does not establish predictive performance across new preparation batches or dates; such transfer would require experiments structured around those sources of variation.

The retained source workbook lists recovered sand, filter catch and wall residue masses. Retrospective author confirmation describes these as dry, tare-corrected measurements from non-overlapping collections, but the correction applied to the recovered sand field is unspecified and a complete dry mass, tare and collection boundary ledger is unavailable. These totals describe collected material records; they are not a closed or component-resolved balance and are not substituted for missing film mass or used to quantify polymer loss. A component-resolved assessment would separately quantify bed-recovered films, outlet films, polymer-containing fines in filters and on walls, sand-associated residues, and water washing solids and dissolved fractions where relevant. Inputs, blanks, drying stages and collection boundaries would need to be matched. Polymer, contamination and sand balances should be evaluated separately; an unassigned difference is not a measured polymer loss.

Downstream material quality requires complementary characterization. Temperature rising elution fractionation (TREF) can quantify PP contamination in recycled PE [42], while PP contamination and compatibilization affect the mechanical response of recycled high-density polyethylene (HDPE) [43]. Molecular and rheological measurements address degradation [44]; processing conditions and antioxidants affect visual properties [45], and seasonal composition can influence recycled PP performance [46]. These are extensions beyond the measured endpoints, which do not establish molecular integrity or suitability for a particular product. Resource inputs and output quality are also needed for a process-level comparison, as illustrated by integrated flexible plastic recycling evaluations [47].

The computational checks establish agreement among the supplied numerical records and calculated outputs. They do not resolve missing acquisition chronology, preparation batch links or retrospectively described procedures. These limitations constrain independent verification and transfer of the findings beyond the retained experimental configurations.

## 5. Conclusions

The count-matched weighing criterion retained 26 of 84 model film runs and left ten of 21 groups without an eligible apparent gravimetric observation. Eligibility differed between dry cleaning and the laboratory stirred water reference, so the mass subset describes count-complete recovered populations rather than all tested conditions. In BBD-R02, one mean mass clean piece corresponded to a 19.5 percentage point contribution to the unrestricted index, quantifying why a missing piece cannot be assigned to contaminant removal.

Optical recovery remained calculable for all runs, with normalization denominators of 68.0–76.2 percentage points and a conditional sampling uncertainty component of 0.33–0.99 percentage point. Separately selected optical samples and stage-specific PE blanks characterize different sources of variation in the measurements. Reporting each endpoint alongside its sampled or weighed population makes clear which laboratory comparisons are supported and how incomplete film recovery affects their interpretation. These conclusions concern the supplied numerical records and measured endpoint populations; incomplete acquisition and material batch documentation limits independent verification of the underlying experimental history.

## Figures and Tables

**Table 1:** Model film datasets and endpoint-specific observation counts.

Dataset	Time (s)	*n*_Total_	$n_{I_{t}}$	$n_{R_{N}}$	$n_{I_{m,\mathrm{app}}}$	Gravimetric Eligibility (%)
Three-factor design	120	17	17	17	6	35.3
Confirmation settings	120	9	9	9	2	22.2
Other material configurations	120	9	9	9	4	44.4
Constant flow reference	120	9	9	9	5	55.6
Nominal RH settings	120	6	6	6	0	0.0
Higher nominal PE loading	120	3	3	3	0	0.0
Records labelled BOUND-OIL-CYCLE	600	10	10	10	0	0.0
Records labelled BOUND-OIL-RESTORE	600	3	3	3	0	0.0
Dry cleaning/stirred water reference	600	18	18	18	9	50.0
Total	120/600	84	84	84	26	31.0

*n* denotes recorded cleaning runs contributing to an endpoint, not individual optical readings. Sand history labels are retained as source identifiers; batch continuity is not independently established. The 10 and three records under these labels remain descriptive observations and are not treated as independent sand batches. Box–Behnken design (BBD) observations represent different design settings. The shared VAL-COMP observations are counted once. Gravimetric eligibility requires matched input and weighed counts and consistent weighing scope; an ineligible mass endpoint leaves optical and count observations available. RH denotes relative humidity.

**Table 2:** Duration-matched laboratory observations at 600 s.

Material	Protocol	*n*_Total_	*I_t_* (%)	*I_t_* Conditional 95% Interval (%)	*R_N_* (%)	*I_m_*_,app_ *n*_Valid_	*I_m_*_,app_ (%)
PE	Dry cleaning	3	97.3 ± 0.2	96.8–97.8	99.2 ± 0.0	0	EX
PE	Stirred water reference	3	98.4 ± 0.3	97.7–99.2	99.7 ± 0.5	2	99.2 ± 0.2
PP	Dry cleaning	3	96.4 ± 0.5	95.0–97.8	99.4 ± 0.5	1	98.9
PP	Stirred water reference	3	98.6 ± 0.7	97.0–100.2	100.0 ± 0.0	3	98.9 ± 0.4
PET	Dry cleaning	3	95.3 ± 0.2	94.7–95.9	99.0 ± 0.0	0	EX
PET	Stirred water reference	3	97.2 ± 0.2	96.7–97.8	100.0 ± 0.0	3	98.6 ± 0.2

Values are means ± sample SD unless a single eligible value is shown. EX indicates no eligible apparent gravimetric observation and does not denote zero. The *I_t_* intervals are within-condition *t* intervals conditional on independent, approximately normal run-level variation. A single value has no SD or interval. Zero observed count SD reflects identical recorded counts, not zero population uncertainty. Additional endpoint-specific group intervals are in R1_Group_CI; the five optical Welch contrasts are summarized in Table S24 and R1_Contrasts. Intervals are not clipped at 0% or 100%; extension beyond those values reflects the parametric interval construction, not an additional observed value.

## Data Availability

The retained numerical records, endpoint calculations and analysis worksheets are provided in Supplementary_Data.xlsx. Supporting_Files.zip contains the source workbooks, optical repeat reading and PE blank records, native SEM–EDS files, analysis and plotting scripts, required inputs, and the prior study crosswalk. Table S19 identifies the evidence available for each dataset and distinguishes derived results from retrospective author confirmations and unavailable acquisition information. The supplied files support recalculation; they do not provide complete instrument-native records, acquisition chronology or preparation batch histories for the cleaning campaign. Figure files are supplied in Figures.zip.

## Supplementary Materials

The following supporting information can be downloaded at: https://www.mdpi.com/article/10.3390/polym18202593/s1, Supplementary_Materials.docx contains Sections S1–S13, Tables S1–S25 and Figures S1–S4, including detailed methods and the dataset evidence table. Supplementary_Data.xlsx provides observations, endpoint eligibility and analysis worksheets, including R1_Provenance, R1_Material_Record, R1_Eligibility, R1_Missing_Film, R1_Optical_Uncertainty, R1_Group_CI, R1_Contrasts and R1_Blank_Scale. Supporting_Files.zip provides the analysis and plotting scripts, necessary inputs, optical repeat-reading and PE blank records, control-target illustration, prior-study crosswalk and native SEM–EDS files. Figure files are provided in Figures.zip. Reference [30] is cited in the Supplementary Materials.
