# Supplementary material for: Film Recovery Constraints on Optical and Gravimetric Assessment of Pulsed Gas–Solid Cleaning of Model Plastic Films
Source: Polymers (Basel). 2026 Oct 21;18(20):2593. doi: 10.3390/polym18202593 (PMC13708878; doi:10.3390/polym18202593)
Supplement: Supplementary file 1 [file polymers-18-02593-s001.zip › polymers-4616367_Supplementary_Materials_tracked.pdf]

Supplementary Materials

Film Recovery Constraints on Optical and Gravimetric Assessment of Pulsed Gas–Solid Cleaning of Model Plastic Films

Supplementary_Data.xlsx contains the measurements, endpoint eligibility and derived analyses. Supporting_Files.zip provides source workbooks, calculation and plotting code, optical-validation and PE-blank data, control-logic documentation, the prior-work comparison and native SEM–EDS files. The cleaning run is the analysis unit; the film population represented by each endpoint is specified below. References follow the main article.

S1. Data sources and relationship to prior work

The dataset comprises 84 unique model-film Run_IDs: 75 dry-cleaning runs and nine laboratory stirred-water reference runs. VAL-COMP contributes to several comparisons but is counted once. S0_Provenance links each run to the mass, count and optical source tables. Table S1 compares the present records and methods with Jin et al. [30] (pp. 305–315) and its four-figure supplement. The authors identify the retained records as a separate experimental series collected using the established apparatus and endpoint definitions. Earlier run-level records and a complete acquisition chronology were unavailable for direct matching. Dataset independence is therefore an author attestation, not an independently verified result; method reuse and derived analyses are distinguished in Tables S1 and S19.

The retained source tables permit recalculation but are not instrument-native acquisition files. Complete acquisition dates, preparation-batch histories and some instrument records are unavailable. Table S19 distinguishes retained records, derived quantities, retrospective author confirmations and evidence that cannot be independently verified. Waste-PE ratios remain separate in Section S9. Optical recovery uses the three state means, film-count recovery uses N_{m2}/N_{in}, and apparent gravimetric analysis requires matched input and weighed-film counts and the stated mass scopes. These rules yield 84 optical and count endpoints, with 26 eligible and 58 excluded gravimetric observations. Numerical agreement does not establish verification of the historical acquisition process.

Table S1. Relationship between the earlier study and the present work.

Relationship	Earlier study [30]	Present records and location	Evidence and limitation
Reused apparatus	Gas–solid fluidized-bed apparatus: p. 306 and Fig. 1, p. 307.	The established platform underlies the reported conditions (Section S3; Table S20).	Published apparatus description supports method comparison. Apparatus reuse is retrospectively confirmed; historical controller exports are unavailable.
Inherited methods and conditions	HDPE bag film, 0.04 mm thick and approximately 3 cm square; triangular pulses at 1 and 1.67 Hz. Washing used 20 g in 300 mL water for 15 min, followed by three rinses with 500 mL water (pp. 306–307, 310).	Reported PE/PP/PET center thicknesses are 30/32/25 μm; prescribed targets are sinusoidal; stirred-water reference approximately 3 g in 50 mL for 600 s (Sections S3 and S4).	Present conditions are retained descriptions/settings. Thickness evidence differs by material (Table S20); instantaneous waveform and acquisition history are not independently established.
Inherited endpoint expressions	PRI_m1, Eq. (1), p. 307, and PRI_t1, Eq. (3), p. 308.	Algebraically equivalent to the present unrestricted apparent mass and optical indices (Methods 2.4; Section S6).	Equivalence can be checked from the published expressions. No claim to have invented these formulas.

Relationship	Earlier study [30]	Present records and location	Evidence and limitation
Current run records	Published methods report averages of two experiments (p. 307); earlier run-level source tables are unavailable.	84 study-specific Run_IDs linked internally to mass/count and optical source workbooks (S0_Provenance; S1a_Cleaning; S2_Optical).	Authors identify a separate series. Earlier raw records and complete chronology are unavailable for direct matching; independence is author attestation.
Derived analyses	Underlying measurement expressions are inherited as above.	Count-based gravimetric eligibility, missing-film scenarios, optical sampling sensitivity, conditional intervals and PE blank-stage comparisons (Tables S21–S25).	These are calculations from retained records, not newly acquired observations. Computational agreement does not verify historical acquisition.
Separate measurement datasets	The published article identifies an LS162 meter (p. 308).	Reported LS116 measurements; 27 films × (10 same-position + 10 reposition readings) = 540 readings, and 12 PE blank records (Sections S7 and S11).	Repeat-reading timing/specimen links and blank date/batch/run matching are not independently established. Neither dataset is presented as verified contemporaneous campaign validation.
Current figures and tables	Article and SI figures are prior sources; SI Figs S1–S4 compare film size, sand size, temperature and humidity.	Current quantitative figures derive from the retained run records. Figure 5 arranges four native SEM fields with their scales.	Current figure provenance is identified separately from prior published figures. Image metadata do not establish cleaning-Run_ID links.
Waste-PE ratios	Pulsed cleaning of PE on the gas–solid platform.	Section S9 contains 36 ratios from 12 source batches with three subsamples per batch, separate from the 84 model-film runs.	Paired original masses and complete batch histories unavailable. These ratios do not validate the model-film campaign.

The prior article and supplement support method-level comparison. This table and the crosswalk do not replace unavailable earlier run-level records or establish record-by-record dataset independence.

[30] Jin, D.; Yu, C.; He, W.; Hu, X.; Zhu, H.; Li, G. Dry cleaning process and mechanism of gas–solid fluidized bed for waste film plastics. J. Mater. Cycles Waste Manag. 2025, 27, 305–315. https://doi.org/10.1007/s10163-024-02111-5

S2. Masses, film counts, and calculations

Worksheet S1a_Cleaning retains masses, film counts, state-mean transmittances and endpoint formulas for all 84 model-film runs. Apparent gravimetric change, $I_{m,\mathrm{app}}$, is summarized for the 26 runs whose retained masses have the stated scope and whose weighed-film count equals the input count. The remaining 58 unrestricted calculations are retained as diagnostic values and marked EX for the restricted gravimetric analysis; their optical and count endpoints remain available. Matching whole-film counts addresses omitted pieces but cannot exclude abrasion, leaching or retained medium.

The retained protocol defines m_0 and m_1 as the masses of the full input-film set. For dry cleaning, m_2 covers bed-recovered films and excludes separately collected outlet pieces; for the stirred-water reference, m_2 covers the recovered films used for weighing. The same recovered population is assigned to m_2 and m_b after terminal washing. Consistent use of these weighing scopes is retrospective author confirmation, without complete contemporaneous weighing-population logs. Bed-recovered and separately collected outlet pieces are recorded separately. All 75 dry-cleaning records satisfy the whole-piece count balance, but the retained totals do not provide a fragment-level audit. Count agreement does not exclude unobserved fines, partial-film loss or counting uncertainty, and is not a polymer mass yield.

The retained source workbook lists recovered-sand, filter-catch and wall-residue masses. Tare subtraction, dry-mass measurement and non-overlapping collection scopes are retrospective author confirmations; a complete collection, tare and dry-mass ledger is unavailable. The correction applied to the recovered-sand field is unspecified, and the totals do not resolve polymer, contaminant and sand contents. They describe recorded material destinations, not a closed or component-resolved balance, and are not substituted for missing-film mass or used to quantify polymer loss. The sand-transfer limitation is described in Section S3.

S3. Operating conditions and endpoint-specific sample sizes

S1b_Conditions lists film loading, sand mass, film-to-sand ratio, duration, controller settings, recorded mean velocities and nominal RH labels. Parameter selection through an integrated controller's built-in program is retrospective author confirmation. The supplied Control_Logic implementation calculates the documented sinusoidal targets; it is not a contemporaneous controller export. Mean velocity, amplitude and frequency are nominal settings because time-resolved gas-flow records are unavailable. The 60% and 80% RH labels identify nominal environmental configurations. Sand and film moisture were not independently measured or controlled, and no continuous temperature/RH record is available.

The laboratory stirred-water washing reference used approximately 3 g of film in a 100 mL glass beaker with 50 mL of fresh deionized water per run. Mechanical stirring was set to 50 r·min^{-1} for 600 s, followed by drying at 45–50 °C for 24 h without a further rinse. This reference is distinct from terminal ultrasonic washing (Section S10). According to retrospective author confirmation, the 120 s and 600 s observations used different films from the same procurement/preparation batches; they do not form a continuous-treatment trajectory. Energy input and treatment intensity were not measured, and equal duration does not match media, handling or the complete drying chain.

Thirteen 600 s records carry the source labels BOUND-OIL-CYCLE (ten records) and BOUND-OIL-RESTORE (three records). The source notes use repeated-use and post-heating labels; retrospective author confirmation specifies a 600 °C, 5 h treatment. The recorded sand masses could not be reconciled with a verified run-to-run transfer history. These labels identify source records, not a validated continuous sand-batch experiment or 13 independently prepared sand batches. The film masses, counts and optical readings remain in the 84-run dataset and are reported descriptively, without inference about cumulative use, heating effects or regeneration.

A conditional continuity screen compared each next recorded sand mass with the preceding sand mass plus the entire preceding contaminated-film mass. In Source_Workbooks/2-不同实验组记录.xlsx, sheet 04_运行原始记录, rows 55→56 give 1996.62 g + 3.1421 g = 1999.7621 g, below the next recorded 2003.82 g by 4.0579 g; rows 60→61 give a further excess of 0.2140 g. This screen assumes the same transferred sand batch, comparable dry-mass and tare bases, and no other input. The excesses identify an unresolved mass-basis or transfer-history problem under those assumptions; they are not a complete component balance. The 13 film-endpoint records support descriptive endpoint analysis; the sand-mass records do not establish continuous-batch or heating effects.

According to retrospective author confirmation, film mass and count were the only deliberately changed operating or preparation conditions between the approximately 3 g and 5 g PE configurations. Bed occupancy, shielding and contact frequency were not measured separately; this comparison does not identify a single loading mechanism.

Table S2. Run counts and endpoint-specific valid observations.

Group	n_{total}	$I_{m,app}$ n_{valid}	I_t n_{valid}	R_N n_{valid}	$I_{m,app}$ eligibility (%)
BBD	17	6	17	17	35.3
VAL-HC	3	0	3	3	0.0

Group	n_{total}	$I_{m,app}$ n_{valid}	I_t n_{valid}	R_N n_{valid}	$I_{m,app}$ eligibility (%)
VAL-LE	3	2	3	3	66.7
VAL-COMP	3	0	3	3	0.0
MAT-PP-COMP	3	1	3	3	33.3
MAT-PET-COMP	3	1	3	3	33.3
MAT-MIX-COMP	3	2	3	3	66.7
CTRL-CF-LOW	3	2	3	3	66.7
CTRL-CF-MID	3	3	3	3	100.0
CTRL-CF-HIGH	3	0	3	3	0.0
BOUND-HUM-60	3	0	3	3	0.0
BOUND-HUM-80	3	0	3	3	0.0
BOUND-LOAD-HIGH	3	0	3	3	0.0
BOUND-OIL-CYCLE	10	0	10	10	0.0
BOUND-OIL-RESTORE	3	0	3	3	0.0
DRYWET-PE-DRY	3	0	3	3	0.0
DRYWET-PE-WET	3	2	3	3	66.7
DRYWET-PP-DRY	3	1	3	3	33.3
DRYWET-PP-WET	3	3	3	3	100.0
DRYWET-PET-DRY	3	0	3	3	0.0
DRYWET-PET-WET	3	3	3	3	100.0

Totals are 84 runs, 26 eligible $I_{m,app}$ observations, and 84 observations for each of I_t and R_N. BBD contains 17 distinct design runs, not 17 repetitions of one setting. BOUND-OIL-CYCLE and BOUND-OIL-RESTORE are retained source labels; sand-batch continuity and acquisition order are not independently established. VAL-COMP is counted once. Eligibility percentages use all runs in each listed group as their denominator.

The description of VAL-COMP-01–03 as three separately prepared groups, each loaded once, treated for 120 s and recovered separately, is retrospective author confirmation. Within-condition intervals are conditional on independent run-level variation; film subsamples and repeated optical readings do not add cleaning runs. Complete preparation-batch and acquisition histories are unavailable, and shared preparation, sand history or run order could introduce dependence.

S4. Materials and instruments

Commercial transparent PE, PP and PET films were purchased through an online marketplace. The authors report center-point thicknesses of 30, 32 and 25 μm, respectively, measured with a GREENER digital outside micrometer (0–25 mm range; 0.001 mm display resolution). The PE and PP values are attributed to original-lot remeasurement by retrospective author confirmation; the PET measurement cannot be linked to the original experimental lot. Individual thickness readings, specimen identifiers and measurement dates were not retained. The PET value is a reported material descriptor, not a verified thickness of the tested lot. The 30 ± 2 mm side length is a cutting target. MIX was prepared by equal mass and inherits the evidence limits of its three constituents; no unified MIX thickness is assigned. Initial optical states and contamination

increments are in S6_Initial_State. S1_Geometry contains nominal-geometry diagnostics, not measured density or area-normalized contamination.

The retained Methods identify an LS116 new-version transmittance meter (Shenzhen Linshang Technology Co., Ltd., Shenzhen, China; 0.01 percentage-point display step over 10–100%), an AL204-IC balance (METTLER TOLEDO; 0.0001 g readability), and a KQ3200DE ultrasonic cleaner (Kunshan Ultrasonic Instruments Co., Ltd., Kunshan, China). Model-film weighing as one stable net reading after taring is retrospective author confirmation. Native TIFF metadata identify the Sigma 300 microscope and SE2 detector; the ZEISS manufacturer identity is supported by manufacturer documentation. Table S20 separates the reported specifications from their sources and missing identifiers. Calibration and verification records for the mass and optical instruments are unavailable. Readability and display resolution are not accuracy or uncertainty estimates.

Contamination preparation used campus soil dried at 60 °C for 48 h and edible oil; the cleaning medium was 40–70 mesh quartz sand. The films were tested as commercial material–geometry configurations. Their grades, film-forming methods, molecular-weight distributions and additives were unspecified, and no thickness-matching rationale or identity spectra were available. These comparisons therefore do not isolate polymer chemistry or molecular integrity. Table S20 lists the reported materials and equipment.

S5. Effect of the gravimetric inclusion rule

The count-matching rule retains 26/84 runs and excludes the gravimetric endpoint in ten of 21 groups; three further groups contain one eligible observation. Table S3 preserves unrestricted and eligible-subset values, while Table S21 gives group eligibility and within-group descriptions of included and excluded records. Because eligibility depends directly on whole-film recovery, the selected gravimetric subset favors more complete recovery and cannot represent all runs or all operating conditions. Similar pooled optical means would not remove this selection effect.

Table S3. Availability of endpoint observations and scope of apparent gravimetric calculations.

Group	Total *n*	I_t *n*	R_N *n*	$I_{m,\mathrm{app}}$ *n*	Unrestricted mean (%)	Restricted mean (%)
BBD	17	17	17	6	95.2	93.0
VAL-HC	3	3	3	0	99.5	EX
VAL-LE	3	3	3	2	95.5	95.4
VAL-COMP	3	3	3	0	99.2	EX
MAT-PP-COMP	3	3	3	1	98.8	99.2
MAT-PET-COMP	3	3	3	1	98.2	98.1
MAT-MIX-COMP	3	3	3	2	97.8	97.6
CTRL-CF-LOW	3	3	3	2	73.7	74.0
CTRL-CF-MID	3	3	3	3	82.2	82.2
CTRL-CF-HIGH	3	3	3	0	80.8	EX
BOUND-HUM-60	3	3	3	0	94.2	EX
BOUND-HUM-80	3	3	3	0	84.6	EX
BOUND-LOAD-HIGH	3	3	3	0	87.1	EX
BOUND-OIL-CYCLE	10	10	10	0	Records; see Section S3	EX
BOUND-OIL-RESTORE	3	3	3	0	Records; see Section S3	EX

Group	Total *n*	I_t *n*	R_N *n*	$I_{m,\mathrm{app}}$ *n*	Unrestricted mean (%)	Restricted mean (%)
DRYWET-PE-DRY	3	3	3	0	98.8	EX
DRYWET-PE-WET	3	3	3	2	99.1	99.2
DRYWET-PP-DRY	3	3	3	1	98.8	98.9
DRYWET-PP-WET	3	3	3	3	98.9	98.9
DRYWET-PET-DRY	3	3	3	0	98.4	EX
DRYWET-PET-WET	3	3	3	3	98.6	98.6

Unrestricted calculations describe the recorded mass difference before the count-based restriction. EX marks no eligible gravimetric observation. A group with one eligible value is reported as a single value without SD or a confidence interval. The source-labelled sand-history records retain individual observations without replicate-style aggregation or a verified continuous-use interpretation. Full-precision values are available in S6_Group_Summary and the derived analysis files.

BBD-R02, the first incomplete-recovery run in alphanumeric Run_ID order, illustrates the contribution of an omitted film. Its m_0, m_1 and m_2 are 3.0017, 3.1301 and 3.0032 g, respectively; 120 pieces were input and 119 were weighed. The unrestricted index is 98.8%. At the mean clean-film mass per input piece, one omitted piece corresponds to about 19.5 percentage points of that index. Table S22 shows three BBD-R02 scenarios; complete run–scenario records are provided in Supplementary_Data.xlsx. The scenario mass neither replaces m_2 nor estimates the actual missing-film mass.

For each incomplete-recovery run, the clean-film omission scenario is $q = \alpha(N_{\mathrm{in}} - N_{m2})m_0/N_{\mathrm{in}}$ for $\alpha = 0.5$, 1.0 or 1.5. The scenario index is $I_{\mathrm{scenario}} = I_{\mathrm{raw}} - 100q/(m_1 - m_0)$. These multipliers illustrate sensitivity rather than measured missing masses or confidence limits. Recorded masses and restricted eligibility remain unchanged.

S6. Single-film optical readings and conditional sampling sensitivity

Worksheet S2_Optical in Supplementary_Data.xlsx contains 2268 readings: 84 runs × three states × nine films, with one center reading per selected film. According to retrospective author confirmation, films were mixed and nine films were selected without replacement for each state; difficult-to-measure films were not deliberately excluded. Individual films were not tracked across states. For dry cleaning, post-cleaning films came from the bed-recovered population. No contemporaneous sampling list or recorded randomization procedure was retained, so sampling probabilities and selection bias cannot be independently reconstructed. Mixing alone does not document probability sampling. Clean- and contaminated-state candidate populations and batch links are incompletely recorded. The nine readings are subsamples; the cleaning run remains the analysis unit.

State means are denoted $\overline{T}_0$, $\overline{T}_1$ and $\overline{T}_2$ for clean, contaminated and post-cleaning films. The optical recovery index is calculated from these means, with $D = \overline{T}_0 - \overline{T}_1 > 0$. At $I_t = 0$%, the post-cleaning and contaminated means coincide; at 100%, the post-cleaning mean equals the chosen clean reference. A value above 100% would indicate a higher mean than that reference and could reflect selection, surface-optical differences or reading variation; it would not mean removal of more than 100% of contamination. Values are not clipped to 0–100%. The authors retrospectively identify the optical measurements as having followed the stated drying procedures. State-specific cooling times and equilibration logs were not retained; equivalent moisture equilibration across the optical states is therefore not independently verified.

$$I_t = 100\frac{\bar{T}_2 - \bar{T}_1}{\bar{T}_0 - \bar{T}_1} \quad \text{(S1)}$$

Each of the 252 run–state sets is summarized by its nine-film mean, sample SD, standard error $s/\sqrt{9}$ and range. The observed SD includes selected-film heterogeneity and reading variation. Conditional propagation through I_t assumes independent state means: with $g_0 = -I_t/D$, $g_1 = (I_t - 100)/D$ and $g_2 = 100/D$, $u_{\text{sampling}}^2(I_t) = g_0^2 s_0^2/9 + g_1^2 s_1^2/9 + g_2^2 s_2^2/9$, and $u_{\text{sampling}}(D) = \sqrt{s_0^2/9 + s_1^2/9}$. This describes a sampling component; undocumented calibration, shared drift, sampling-frame effects and batch effects are outside the calculation. The without-replacement procedure is retrospectively reported; candidate-population sizes and contemporaneous selection records are unavailable, so no finite-population correction is applied and unrecorded selection effects remain outside this conditional calculation.

Across the retained runs, D ranges from 68.0 to 76.2 percentage points. The conditional sampling component for I_t is approximately 0.33–0.99 percentage points. Table S23 provides the 252 state summaries and 84 run-level sensitivities, including independent within-state resampling intervals. Display-rounding sensitivity remains separate: each state mean is perturbed by ±0.005 percentage points and all eight sign combinations are evaluated. Its largest absolute index change is approximately 0.0147 percentage points, and the signs of the examined group contrasts were unchanged across the perturbation corners. This small rounding effect concerns the present large-denominator data; it supplies neither a total uncertainty bound nor a validated threshold for lightly contaminated films.

Conditional resampling independently draws nine readings with replacement within each run–state set for 10,000 iterations, using a PCG64 generator and seed 20261008. The 2.5th and 97.5th percentiles of I_t, with linear quantile interpolation, form a conditional sampling interval. States are not paired, and resampling iterations are not cleaning experiments. If a denominator is small relative to sampling or measurement variation, the state transmittances and an independently checked reference are needed; these data do not define a universal validity threshold.

S7. Separate optical repeat-reading dataset

A separate optical repeat-reading dataset comprised 27 films, with three films for each PE, PP and PET material–state combination. Each film was read ten times at the same position and ten times after removal and repositioning, yielding 270 readings per mode. The retained CSVs contain no acquisition timestamps or links between validation specimens and cleaning Run_IDs. These measurements characterize within-film reading and repositioning variability; they do not establish contemporaneous validation of the 84-run campaign or increase its number of cleaning runs. Its timing relative to the cleaning campaign cannot be independently established from the retained files. The readings are in S2_Optical_Validation and the two original CSVs in Optical_Validation.

Across the nine material–state combinations, mean within-film SDs are 0.0218–0.0409 percentage points at a fixed position and 0.1647–0.2370 after repositioning, with ratios of 4.214–9.467. This position variance is not added again to the nine-film variance, which already includes reading variation. A transmittance-position component and a normalized run-level I_t contrast represent different quantities and cannot be compared directly as a reliability test. These measurements characterize repeatability and repositioning variability; they do not establish absolute accuracy.

S8. SEM–EDS observations and elemental results

The fields PE1–PE4 represent clean, mud–dust-contaminated, stirred-water-reference and dry-cleaned PE, respectively. Gold sputter coating and mechanical clamping are retrospective author confirmations. One field per state is analyzed here; additional original fields were unavailable for analysis. Cleaning-Run_ID links and the field-selection procedure are undocumented. Native image bars show 1000× magnification, 15 kV, SE2 detection, 10 μm scale bars and working distances of 7.7, 7.7, 7.9 and 7.8 mm. The images are presented with their native scales; full-image dimensions do not define the physical EDS analysis area.

Table S4. SEM–EDS sample states and original files.

Sample	State	Original SEM file	EDS report	WD (mm)	Image-bar date
PE1	Clean	1-PE1- M.tif	1-PE1-M.docx	7.7	30 July 2026
PE2	Mud–dust-contaminated	2-PE2-M.tif	2-PE2-M.docx	7.7	30 July 2026
PE3	Stirred-water reference	3-PE3-M.tif	3-PE3-M.docx	7.9	30 July 2026
PE4	Dry-cleaned	4-PE4-M.tif	4-PE4-M.docx	7.8	30 July 2026

Original filenames and state identifiers are preserved. The TIFF headers identify the Sigma 300 model, serial Sigma 300-8211011409, with SEM software V07.00.00.00. The image bars and spectrum headers give 30 July 2026 as the microscopy date; that date does not identify the preceding cleaning run or preparation batch. The EDS spectrum headers report magnification values of 1000 for PE1–PE3 and 1169 for PE4, distinct from the 1000× SEM image labels. These fields do not establish identical SEM and EDS analysis areas.

The authors retrospectively identify the EDS acquisition as third-party laboratory work. Tables S5–S8 report the eight listed elements C, O, Na, Mg, Al, Si, K and Ca. Mass and atomic fractions are normalized software outputs. The samples were gold-coated, but Au was not included in the reported normalization; these values therefore describe the listed-element total rather than the complete analyzed composition. The quantification algorithm and coating correction were unspecified. Reported Sigma values represent counting or fitting uncertainty, not variation across independent specimens, and reported zeros do not establish elemental absence or detection limits. The results describe local elemental observations alongside the SEM fields; they are not used to estimate bulk composition, PE purity or a quantitative ranking of cleaning routes.

Table S5. EDS results for clean PE (PE1).

Element	Mass fraction (%)	Mass-fraction Sigma	Atomic fraction (%)
C	99.05	0.11	99.31
O	0.86	0.11	0.65
Na	0.00	0.02	0.00
Mg	0.02	0.01	0.01
Al	0.02	0.01	0.01
Si	0.05	0.01	0.02
K	0.00	0.01	0.00
Ca	0.00	0.01	0.00

Software-reported normalized fractions; illustrative only. Sigma is a report output, not between-specimen SD.

Table S6. EDS results for mud–dust-contaminated PE (PE2).

Element	Mass fraction (%)	Mass-fraction Sigma	Atomic fraction (%)
C	68.64	0.26	76.39
O	24.32	0.23	20.32
Na	0.09	0.02	0.05

Element	Mass fraction (%)	Mass-fraction Sigma	Atomic fraction (%)
Mg	0.32	0.01	0.18
Al	1.90	0.03	0.94
Si	3.75	0.04	1.78
K	0.57	0.02	0.20
Ca	0.41	0.02	0.14

Software-reported normalized fractions; illustrative only. Sigma is a report output, not between-specimen SD.

Table S7. EDS results for PE from the stirred-water reference (PE3).

Element	Mass fraction (%)	Mass-fraction Sigma	Atomic fraction (%)
C	90.08	0.17	92.93
O	8.20	0.17	6.35
Na	0.05	0.02	0.03
Mg	0.05	0.01	0.02
Al	0.35	0.01	0.16
Si	0.89	0.02	0.39
K	0.10	0.01	0.03
Ca	0.29	0.02	0.09

Software-reported normalized fractions; illustrative only. Sigma is a report output, not between-specimen SD.

Table S8. EDS results for dry-cleaned PE (PE4).

Element	Mass fraction (%)	Mass-fraction Sigma	Atomic fraction (%)
C	97.25	0.13	98.01
O	2.47	0.13	1.87
Na	0.02	0.02	0.01
Mg	0.00	0.01	0.00
Al	0.08	0.01	0.04
Si	0.14	0.01	0.06
K	0.02	0.01	0.01
Ca	0.02	0.01	0.01

Software-reported normalized fractions; illustrative only. Sigma is a report output, not between-specimen SD.

S9. Waste-PE terminal-wash observations

The waste-PE dataset comprises 12 source batches, each represented by three separately treated nominal 3.0 g subsamples. The retained protocol describes combining material from several positions in each stockpile before industrial cleaning, followed by subsample allocation and 120 s treatment at nominal mean velocity 0.68 m·s^{-1}, amplitude 0.12 m·s^{-1} and frequency 1.50 Hz. Table S9 preserves all 36 terminal-wash ratios and their original source labels. Across-source summaries use the 12 batch means rather than treating the 36 within-batch results as independent sources.

PE-01–PE-04 denote logistics stretch-film waste, PE-05–PE-08 commercial packaging-film waste and PE-09–PE-12 separated courier-bag waste. R1–R3 identify the three recorded treatments within each batch. The documented protocol used all recovered films from each subsample for TW-01 (Table S10). Paired dry masses, run-level weighing populations and intermediate weights are unavailable. The ratios are therefore descriptive observations and are not used to validate model-film cleaning efficacy or to infer missing masses.

Table S9. Terminal-wash mass-change ratios (R_d) for 12 waste-PE source batches.

Source batch	Source category	Loading per run (g)	Time (s)	R_d, R1 (%)	R_d, R2 (%)	R_d, R3 (%)	Mean R_d (%)	Within-batch SD (percentage points)
PE-01	logistics stretch-film waste	3.0	120	0.880	0.910	0.890	0.893	0.015
PE-02	logistics stretch-film waste	3.0	120	0.930	0.950	0.920	0.933	0.015
PE-03	logistics stretch-film waste	3.0	120	0.840	0.870	0.860	0.857	0.015
PE-04	logistics stretch-film waste	3.0	120	0.960	0.980	0.970	0.970	0.010
PE-05	commercial packaging-film waste	3.0	120	0.790	0.820	0.810	0.807	0.015
PE-06	commercial packaging-film waste	3.0	120	0.900	0.940	0.920	0.920	0.020
PE-07	commercial packaging-film waste	3.0	120	1.010	1.030	1.020	1.020	0.010
PE-08	commercial packaging-film waste	3.0	120	0.860	0.880	0.900	0.880	0.020
PE-09	separated courier-bag waste	3.0	120	0.970	0.990	0.980	0.980	0.010
PE-10	separated courier-bag waste	3.0	120	0.820	0.850	0.830	0.833	0.015
PE-11	separated courier-bag waste	3.0	120	1.000	1.040	1.020	1.020	0.020
PE-12	separated courier-bag waste	3.0	120	0.910	0.930	0.950	0.930	0.020

Three separately treated nominal 3.0 g subsamples are recorded per batch. Means and sample SDs summarize the retained within-batch ratios; displayed precision does not establish measurement uncertainty.

The recorded ratios range from 0.790% to 1.040%; batch means range from 0.807% to 1.020%. The mean of the 12 batch means is 0.920%, with an across-batch sample SD of 0.069 percentage points. Each source category contains four batches. Pre-cleaning ratios and paired masses are unavailable, precluding removal-efficiency estimates and supported source-category rankings.

S10. Terminal washing and operational mass responses

Table S10 describes the retained terminal ultrasonic-washing protocol TW-01; it is not a complete run-specific drying, cooling or weighing log. Model-film results are based on paired endpoint masses; waste-PE results are available only as derived ratios. Section S11 separates the PE blank stages and their relevance to the pre-terminal gravimetric endpoint.

Table S10. Ultrasonic terminal-washing procedure TW-01.

Step	Operation
Sample scope	Model films: the recovered films included in m_2. The recorded waste-PE protocol specified the films recovered from each nominal 3.0 g subsample; run-level weighing-population records are unavailable.
Vessel and water	A 100 mL glass beaker containing 60 mL of deionized water.
Ultrasonic treatment	KQ3200DE cleaner; 40 kHz; 100% power setting. The reported nameplate input power is 150 W; this is not measured acoustic power in the liquid.
Duration and water replacement	Two cycles of 180 s each. All 60 mL of water was replaced between cycles. No additional rinse was performed.
Temperature	Heating was switched off and treatment started with water at room temperature. Actual liquid temperature was not recorded.
Drying and weighing	The reported protocol specifies drying at 60 °C for 4 h, cooling in a desiccator for 30 min and weighing, followed by 1 h drying increments with the same cooling/weighing sequence. Run-specific intermediate logs are unavailable.
Constant-mass criterion	Constant mass was defined as a difference below 0.1% of sample mass between successive weighings. The PE blank series includes 4, 5 and 6 h readings, without corresponding cooling times or a stopping-point record; these readings alone do not establish constant mass. Model-film m_2 and m_b refer to the same recovered film set.
Endpoint data	Model-film data include m_2 and m_b, whereas waste-PE data comprise R_d ratios. Intermediate constant-mass sequences are unavailable for both sample types. The separate PE blank series contains measurements at several drying stages.

TW-01 is a retained protocol description. Applying the same recovered-film scope is retrospective author confirmation; intermediate drying, cooling and tare records do not independently verify its execution for each run. A stated stopping rule is not evidence that constant mass was attained.

Intermediate drying weights for the model films and waste PE are unavailable, so attainment of the stated constant-mass criterion cannot be assessed from the endpoint masses.

For model films, the operational terminal-wash mass-change ratio, R_d, and apparent mass deficit, L_{app}, are defined as follows:

$$R_d = 100\frac{m_2 - m_b}{m_2} \quad \text{(S2)}$$

$$L_{\text{app}} = 100(1 - \frac{m_b}{m_0}) \quad \text{(S3)}$$

For model films, m_2 and m_b refer to the same recovered set but follow different drying procedures: 45–50 °C for 24 h before m_2, and the reported 60 °C procedure before m_b. R_d combines terminal-washing, drying,

handling and possible polymer contributions. L_{app} compares m_b with full-input m_0 and is restricted to count-matched sets; it does not isolate polymer loss. Figure S1 presents R_d with the corresponding sample scopes. Blank stages are examined separately below rather than subtracted from primary endpoint values.

S11. PE clean-film blanks

The 12 retained PE blank records comprise four conditions with three records each: A, drying only; B, terminal washing only; C, 120 s gas–solid handling followed by terminal washing; and D, 600 s gas–solid handling followed by terminal washing. The blank file does not identify acquisition dates, material-batch identifiers or matched cleaning Run_IDs. The author's description of concurrent, same-batch operation is retrospective confirmation, not independently verified matching. The records are used as a separate, stage-specific PE sensitivity dataset. Blank_Validation/Blank_Mass_Validation_Raw.csv and S5_Blank_Validation provide initial, post-45–50 °C, pre-terminal, 60 °C (4, 5 and 6 h), final and count values where recorded. Pre-terminal, terminal-wash and complete-sequence changes are calculated separately; primary endpoints are not corrected by subtracting blank means.

Table S25 separates pre-terminal change b_{pre} = m_after_45_50C_24h − m_initial, terminal change $b_{terminal}$ = m_final − m_before_terminal_wash, and total change b_{total} = m_final − m_initial. The C pre-terminal changes are −1.4, −1.1 and −1.7 mg; the D values are −1.8, −1.5 and −2.0 mg. Mean total changes are −1.17, −2.20, −4.13 and −5.40 mg for A–D. All 12 records retain 120 of 120 pieces and report no blowout. Total values include stages after m_2 and are not used to correct $I_{m,\mathrm{app}}$.

The 1.1–1.7 mg absolute pre-terminal C-blank range corresponds to approximately 0.82–1.49 percentage points when divided by the apparent contamination increments of the 13 eligible PE 120 s runs. This run-specific scale scenario is $\Delta I_{blank} = 100|b_{pre}|/(m_1 - m_0)$. For signed scenarios, $I_{scenario} = I_{raw} + 100b_{pre}/(m_1 - m_0)$, so a negative blank change lowers the index. Stage alignment does not establish run-level, batch-level or temporal matching. A group-mean correction would also require transferability of the stage-specific blank response to contaminated films and propagation of its uncertainty; one-to-one run pairing is not inherently required. Transferability has not been established, and dates, batch identifiers and blank-to-Run_ID links are unavailable. PP, PET and MIX have no matching blanks, and 600 s dry-cleaned PE has no eligible gravimetric value. The values are used to assess the scale of possible blank contributions, without a universal subtraction from primary observations.

S12. Data dictionary, missing-value codes, and numerical reporting

Table S11. Variable definitions, units, and analysis levels.

Variable	English name	Unit	Level	Data type	Definition and scope
Run_ID	Run identifier	None	Run	Recorded identifier	Unique key. Shared reference runs are not counted more than once.
m_0	Clean-film mass	g	Run	Recorded measurement	Mass of all input model-film pieces before contamination. The weighing-population scope is retrospectively confirmed; contemporaneous population logs are incomplete.

Variable	English name	Unit	Level	Data type	Definition and scope
m_1	Contaminated-film mass	g	Run	Recorded measurement	Mass after contamination, using the same film set as m_0. The weighing-population scope is retrospectively confirmed; contemporaneous population logs are incomplete.
m_2	Recovered-film mass after cleaning	g	Run	Recorded measurement	For dry cleaning, bed-recovered films only. For the laboratory stirred-water reference, the recovered films used for weighing. The weighing-population scope is retrospectively confirmed; contemporaneous population logs are incomplete.
m_b	Mass after terminal washing	g	Run	Recorded measurement	Mass of the same recovered films after the reported 60 °C terminal procedure; intermediate constant-mass readings are unavailable. The weighing-population scope is retrospectively confirmed; contemporaneous population logs are incomplete.
N_{in} / N_{m2}	Input film count / count included in m_2	Films	Run	Recorded count / scope correspondence	Counts define the input and weighed-film scopes. Their ratio is not a mass yield.
$I_{m,app}$	Apparent gravimetric change	%	Run	Calculated	$100(m_1 - m_2)/(m_1 - m_0)$, included only when the film counts and mass scopes meet the stated rule.
I_t	Optical recovery index	%	Run	Calculated	$100(\overline{T}_2 - \overline{T}_1)/(\overline{T}_0 - \overline{T}_1)$, using the mean of nine single-film center readings for each state.
R_N	Film count recovery	%	Run	Calculated	$100N_{m2}/N_{in}$.
R_d	Operational terminal-wash mass-change ratio	%	Run	Calculated / retained result	Operational terminal-wash mass ratio, $100(m_2 - m_b)/m_2$; waste-PE entries are recorded terminal-wash ratios summarized by source batch.
L_{app}	Apparent mass deficit	%	Run	Calculated	$100(1 - m_b/m_0)$, restricted to count-matched recovered-film populations; drying and other mass-change contributions remain unresolved.
$\overline{u}_{set}$ / a_{set} / f_{set}	Mean gas-velocity / amplitude / frequency setpoint	$m \cdot s^{-1}$ / $m \cdot s^{-1}$ / Hz	Run	Recorded setting	Control settings, not measured instantaneous gas-flow waveforms.
n_{total} / n_{valid}	Total runs / valid endpoint observations	Runs	Group	Count	Eligibility is evaluated separately for each endpoint.

Variable	English name	Unit	Level	Data type	Definition and scope
SD	Sample standard deviation	Same as variable	Run group / source batch	Calculated	Uses denominator $n-1$; SD is in percentage points for percentage endpoints. Film subsamples do not increase the independent-run count.
$\overline{T}_0$ / $\overline{T}_1$ / $\overline{T}_2$	Mean clean / contaminated / post-cleaning transmittance	%	State within run	Calculated mean	Arithmetic mean of the nine single-film center readings for the specified state.
N_blowout_observed	Observed blown-out film count	Films	Run	Observed count	Separately recorded outlet-piece count, excluded from dry-cleaning m_2. Retained totals do not audit fragments or exclude partial-film loss, duplicate-count uncertainty or unobserved fines.
sand_mass_g	Sand mass	g	Run	Recorded measurement	Retained sand-mass field; dry/tare basis and run-to-run transfer history are not independently established. Source labels do not verify one continuous sand batch (Section S3).
film-to-sand ratio	Clean-film loading per sand mass	g kg^{-1}	Run	Calculated	Clean-film mass divided by sand mass, with the sand mass expressed in kilograms.
u_recorded_mean_m_s	Recorded mean gas velocity	m·s^{-1}	Run	Recorded value	A retained mean value; not a time-resolved gas-flow waveform.
time_s	Cleaning duration	s	Run	Recorded condition	Duration of the cleaning run, distinct from terminal-washing duration.
Optical verified	Optical evidence flag	None	Run	Yes / No	Yes enables calculation of I_t from the three recorded state means, subject to valid ranges and a positive $\overline{T}_0 - \overline{T}_1$ denominator.
Counts verified	Count evidence flag	None	Run	Yes / No	Yes enables count-based calculations from the recorded input and recovered-film counts, subject to non-negative integer and population checks.
Masses verified	Mass evidence flag	None	Run	Yes / No	Yes enables the unrestricted apparent mass calculation from m_0, m_1 and m_2, subject to positive masses and $m_1 > m_0$.
Terminal verified	Terminal endpoint evidence flag	None	Run	Yes / No	Yes enables calculation of R_d from positive m_2 and m_b values for the linked recovered-film population.

Variable	English name	Unit	Level	Data type	Definition and scope
Fit optical model	Model inclusion flag	None	Run	Yes / No	Yes only for included BBD design observations; confirmation groups remain excluded.
Acquisition date	Acquisition date	Date	Run	Recorded / missing	Date of acquisition, where available.
Preparation batch	Preparation batch	None	Run	Recorded / missing	Only documented batches are entered; no groups are derived from design identifiers.
Dataset origin	Dataset origin	None	Run	Study provenance	The authors identify the retained records as a separate current series using reused apparatus/methods. Earlier run-level records and complete chronology are unavailable; independence is author attestation, not independently verified matching.

Yes/No flags describe retained fields or stated scopes used in calculation. They do not certify calibration, acquisition history, selection probabilities or independent batches.

Run_ID joins source worksheets and analysis exports. The dictionary defines each variable's unit, population and analytical role. Existing Yes/No evidence flags refer to retained fields and stated scopes used by the calculations; Yes does not verify instrument calibration, acquisition history, sampling probabilities or batch independence. Retrospectively described procedures are distinguished from contemporaneous evidence in Table S19 and S0_Verification. Blank or NR/NV entries do not imply that an unrecorded procedure occurred or was verified.

Table S12. Missing-value and endpoint-exclusion codes.

Code	Meaning
NA	Not applicable.
NR	Not recorded or not retained.
NV	The available records are insufficient for independent verification.
EX	Excluded from a specified analysis under the stated inclusion rule; the code does not invalidate the underlying recorded data.

Blank source cells and missing-value codes are not zeros. EX is endpoint-specific exclusion, NR denotes records or details not retained, and NV denotes insufficient evidence for independent verification. NA denotes inapplicability.

Calculations retain full input precision. Descriptive indices, SDs and conditional intervals are generally displayed to one decimal place; raw masses retain recorded digits, EDS outputs retain two decimals, waste-PE ratios retain three, and model coefficients remain available at greater precision in the workbook. A single eligible observation has no SD or confidence interval; zero observed count dispersion does not establish zero population uncertainty.

For a repeated within-condition group with $n \geq 2$, a conditional 95% mean interval is mean ± $t_{0.975,n-1}$SD/$\sqrt{n}$; n = 3 uses $t \approx 4.3027$. Each endpoint uses its eligible run count. Zero eligible values are EX, and one value has no SD or interval. The BBD factor design and the 13 source-labelled sand-history records are not treated as replicated homogeneous groups. Zero observed count dispersion can give zero-width calculated limits without establishing zero population uncertainty; a one-piece count increment is 100/N_{in} percentage points.

Five specified optical contrasts use unpaired Welch intervals: stirred-water minus dry-cleaning for PE, PP and PET; nominal 80% minus 60% RH; and nominal 5 g minus 3 g PE loading. With $SE_\Delta = \sqrt{s_A^2/n_A + s_B^2/n_B}$, Welch degrees of freedom are $(s_A^2/n_A + s_B^2/n_B)^2$ divided by $[(s_A^2/n_A)^2/(n_A-1) + (s_B^2/n_B)^2/(n_B-1)]$. The interval is the mean difference ± $t_{0.975,\nu}SE_\Delta$. These exploratory intervals assume independent runs and approximately normal run-level variation; they do not resolve unavailable batch histories or constitute multiplicity-adjusted, causal or equivalence tests.

S13. Descriptive optical models and confirmation intervals

The same 17 Box–Behnken design observations were used for three ordinary least-squares models: linear (4 parameters), additive quadratic without interactions (7), and full quadratic (10). All nine confirmation runs were excluded from fitting. The additive quadratic model provides the simpler descriptive account; the full model is retained as a sensitivity comparison. No fitted maximum is interpreted as a validated optimum. The exclusion of confirmation runs from fitting does not itself establish independence of their preparation batches from the design runs. Run dates and complete preparation-batch links remain unavailable, so batch-held-out validation was not performed. Factors were coded as follows:

$$X_1 = \frac{\bar{u}_{set} - 0.68}{0.16}, \quad X_2 = \frac{f_{set} - 1.50}{1.00}, \quad X_3 = \frac{a_{set} - 0.12}{0.06} \quad (S4)$$

The nominal factor ranges were 0.52–0.84 m·s^{-1} for mean-velocity setting, 0.50–2.50 Hz for frequency setting, and 0.06–0.18 m·s^{-1} for amplitude setting. The full quadratic form below reduces to the additive quadratic model by omitting interactions and to the linear model by also omitting squared terms:

$$Y = \beta_0 + \sum_{i=1}^{3} \beta_i X_i + \sum_{i<j} \beta_{ij} X_i X_j + \sum_{i=1}^{3} \beta_{ii} X_i^2 \quad (S5)$$

The response Y is I_t in percent; residuals and contrasts are in percentage points. All 17 design responses were used. Tables S13–S15 show the full quadratic sensitivity analysis; Tables S16–S18 compare models, provide additive coefficients and distinguish fitted-mean confidence intervals from future-run prediction intervals. Supporting_Files.zip contains the scripts, execution instructions and numerical outputs. Calculations used Python 3.12.10, NumPy 2.4.6 and SciPy 1.18.0.

Table S13. Full quadratic coefficients for I_t as a sensitivity comparison.

Term	Coefficient	Standard error	t	p	95% confidence interval
Intercept	96.52026	0.42629	226.420	8.654e-15	[95.51225, 97.52827]
X_1	0.82702	0.33701	2.454	0.04385	[0.03012, 1.62393]
X_2	-0.37139	0.33701	-1.102	0.3069	[-1.16829, 0.42552]
X_3	0.47259	0.33701	1.402	0.2036	[-0.32432, 1.26949]
$X_1 \times X_2$	-0.12728	0.47661	-0.267	0.7971	[-1.25428, 0.99971]
$X_1 \times X_3$	-0.20940	0.47661	-0.439	0.6737	[-1.33639, 0.91760]
$X_2 \times X_3$	0.38328	0.47661	0.804	0.4477	[-0.74372, 1.51027]
X_1^2	-3.01655	0.46454	-6.494	0.0003361	[-4.11501, -1.91809]
X_2^2	-2.66855	0.46454	-5.745	0.0007024	[-3.76701, -1.57009]

Term	Coefficient	Standard error	t	p	95% confidence interval
X_3^2	-3.14514	0.46454	-6.770	0.0002601	[-4.24360, -2.04668]

Coded factors are dimensionless. Coefficients, standard errors and confidence limits are in percentage points. Conventional t tests and intervals use 7 residual degrees of freedom and are conditional on the error model. HC3 standard errors in S7_Coefficients assess sensitivity to unequal error variance; they do not incorporate measurement or sampling-systematic uncertainty.

Table S14. Analysis of variance for the full quadratic model.

Source	df	Sum of squares	Mean square	F	p
Model	9	131.98681	14.66520	16.14021	0.000683
Residual	7	6.36029	0.90861	—	—
Lack of fit	3	0.04070	0.01357	0.00859	0.9987
Pure error	4	6.31959	1.57990	—	—
Total	16	138.34710	—	—	—

df, degrees of freedom; dash, not applicable. Sums and means of squares use I_t in percentage units. Full-precision results are in Supplementary_Data.xlsx (worksheet S7_ANOVA).

For the full quadratic model, R^2 = 0.9540, adjusted R^2 = 0.8949, and leave-one-out RMSE = 0.7869 percentage points. The five center observations account for 99.360% of its residual sum of squares. The data do not identify the cause of this concentration of residual variation. The residual standard error is 0.9532 percentage points. Equations (S6) and (S7) give 95% prediction intervals for one future run and the mean of m future runs, respectively, using the selected model's residual degrees of freedom ν:

$$\hat{y} \pm t_{0.975,\nu}\sqrt{\mathrm{MSE}(1+h)} \quad \text{(S6)}$$

$$\hat{y} \pm t_{0.975,\nu}\sqrt{\mathrm{MSE}(1/m+h)} \quad \text{(S7)}$$

Here, $h = x^{\mathrm{T}}(X^{\mathrm{T}}X)^{-1}x$, where x is the model-term vector at the confirmation setting and X is the design matrix. For the 95% confidence interval of the fitted mean, $(1 + h)$ in Equation (S6) is replaced by h. For linear, additive quadratic, and full quadratic models, ν is 13, 10, and 7, respectively. The retained confirmation groups each contain m = 3 runs. These intervals answer different questions and assume independent, normally distributed errors with constant variance.

Table S15. Full quadratic predictions and observed confirmation means.

Confirmation group	Predicted I_t (%)	Observed mean I_t (%)	Observed − predicted (percentage points)	95% prediction interval for mean of 3 runs (%)
VAL-HC	96.199	97.749	1.549	[94.564, 97.835]
VAL-LE	93.918	92.152	-1.766	[92.239, 95.598]
VAL-COMP	96.520	96.828	0.307	[94.874, 98.166]

Each group has three confirmation runs excluded from fitting. Settings (mean velocity, amplitude, frequency) were VAL-HC (0.704 m·s^{-1}, 0.144 m·s^{-1}, 1.60 Hz), VAL-LE (0.592 m·s^{-1}, 0.090 m·s^{-1}, 1.15 Hz), and VAL-COMP (0.680 m·s^{-1}, 0.120 m·s^{-1}, 1.50 Hz). Errors use unrounded values. The displayed intervals refer to future means of three runs.

Under the full quadratic model, the VAL-LE mean (92.152%) lies below the lower prediction limit for a mean of three future runs (92.239%), although every individual confirmation result lies within its wider single-run interval. The additive quadratic model misses both VAL-HC and VAL-LE means and at least one single-run result in each of those groups. Thus, individual coverage cannot be used to claim successful confirmation of a group mean. The full model's Shapiro–Wilk *p* value is 0.000743; its nonsignificant lack-of-fit result (p = 0.9987) does not establish model adequacy.

S7_Diagnostics contains fitted values, residuals, leverage, studentized residuals, and Cook's distance for each model; S7_LOO and S7_Omission contain leave-one-out and deletion sensitivity results. The largest change in fitted I_t after one-run omission was 0.473 percentage points for the additive model and 0.431 for the full model. For the full model, maximum Cook's distance was 0.1022 and maximum leverage was 0.75. These checks describe the retained design and do not establish prediction across dates or batches. Figures S3 and S4 show model comparisons and full-model diagnostics; identifier order does not establish chronology or temporal drift.

Table S16. Comparison of three models fitted to the same 17 design observations.

Model	Parameters	Adjusted R^2	LOO RMSE (pp)	Shapiro–Wilk *p*	Center residual SS (%)
Linear	4	-0.1564	3.2786	0.000211	71.282
Additive quadratic	7	0.9169	0.8861	0.111400	87.918
Full quadratic	10	0.8949	0.7869	0.000743	99.360

The additive model has the higher adjusted R^2, while the full quadratic model has the slightly lower LOO RMSE. Similar confirmation errors and unstable interval coverage do not support a strong predictive preference. A nonsignificant normality test does not establish normality in this small design. pp, percentage points; SS, sum of squares.

Table S17. Coefficients of the additive quadratic descriptive model.

Term	Estimate	SE	HC3 SE	*t*	*p*	95% CI
Intercept	96.52026	0.37916	0.62847	254.564	2.152e-20	[95.67544, 97.36508]
X_1	0.82702	0.29975	0.12807	2.759	0.02016	[0.15913, 1.49491]
X_2	-0.37139	0.29975	0.20741	-1.239	0.2436	[-1.03928, 0.29650]
X_3	0.47259	0.29975	0.22168	1.577	0.146	[-0.19530, 1.14048]
X_1^2	-3.01655	0.41318	0.39118	-7.301	2.599e-05	[-3.93717, -2.09593]
X_2^2	-2.66855	0.41318	0.39118	-6.459	7.268e-05	[-3.58917, -1.74793]
X_3^2	-3.14514	0.41318	0.39118	-7.612	1.815e-05	[-4.06576, -2.22452]

Conventional standard errors, *t* tests and confidence intervals use 10 residual degrees of freedom. HC3 standard errors examine sensitivity to unequal error variance. Neither set includes systematic measurement effects or unresolved batch dependence.

Table S18. Fitted-mean confidence intervals and future-run prediction intervals for all models.

Model and group	Predicted I_t (%)	Fitted-mean 95% CI (%)	Single-run 95% PI (%)	Mean-of-3 95% PI (%)	Mean inside PI
Linear; VAL-HC	92.641	[90.674, 94.608]	[85.532, 99.750]	[88.234, 97.048]	No

Model and group	Predicted I_t (%)	Fitted-mean 95% CI (%)	Single-run 95% PI (%)	Mean-of-3 95% PI (%)	Mean inside PI
Linear; VAL-LE	91.804	[89.219, 94.389]	[84.500, 99.108]	[87.088, 96.519]	Yes
Linear; VAL-COMP	92.365	[90.708, 94.022]	[85.336, 99.394]	[88.087, 96.643]	No
Additive quadratic; VAL-HC	96.198	[95.371, 97.025]	[94.136, 98.261]	[94.830, 97.567]	No
Additive quadratic; VAL-LE	93.933	[93.119, 94.748]	[91.876, 95.991]	[92.572, 95.295]	No
Additive quadratic; VAL-COMP	96.520	[95.675, 97.365]	[94.451, 98.590]	[95.141, 97.900]	Yes
Full quadratic; VAL-HC	96.199	[95.209, 97.190]	[93.737, 98.661]	[94.564, 97.835]	Yes
Full quadratic; VAL-LE	93.918	[92.857, 94.980]	[91.427, 96.410]	[92.239, 95.598]	No
Full quadratic; VAL-COMP	96.520	[95.512, 97.528]	[94.051, 98.989]	[94.874, 98.166]	Yes

Observed means are 97.749% (VAL-HC), 92.152% (VAL-LE), and 96.828% (VAL-COMP). The final column compares each observed mean with its mean-of-three prediction interval, using unrounded limits. CI and PI are conditional model intervals, not measurement-uncertainty bounds. All confirmation records remain excluded from fitting.

Additional supplementary tables

Table S19. Dataset provenance and interpretation limits. Part A: retained evidence, derived quantities and analytical use.

Dataset	Retained file and locator	Instrument-native evidence	Derived quantities and use
84 model-film mass and count records	Source_Workbooks/2-不同实验组记录.xlsx, 04_运行原始记录, rows 2–85; S1a_Cleaning and S0_Provenance	Retained source workbook; not a balance-native acquisition export.	84 optical/count/mass endpoints; count-based gravimetric eligibility; 174 missing-film scenarios. Use: All 84 runs retained; apparent gravimetry used for 26 count-matched records.
2268 single-film optical readings	Source_Workbooks/3-不同实验组别洁净-污染-清洁后的透光率情况.xlsx; S2_Optical and S2_Optical_Long; input/optical_long.csv	Retained optical workbook; no instrument-native campaign export.	252 state summaries; 84 optical indices; propagated sampling components and within-state percentile bootstrap intervals. Use: Conditional variation among selected films; independent state resampling.
Operating conditions and sand-history labels	Source run workbook rows 2–85, especially 55–67; S1b_Conditions and S6_Sand_Sequence	Retained settings and notes; no logged airflow waveform or sand-transfer ledger.	Nominal condition groups; 13 source-labelled film-endpoint records. Sand screen: rows 55→56, 4.0579 g; rows 60→61, 0.2140 g. Use: Retain BOUND-OIL-CYCLE/RESTORE IDs, film masses/counts/optics and all 84-run calculations.

Dataset	Retained file and locator	Instrument-native evidence	Derived quantities and use
Collected-material masses	Source_Workbooks/2-不同实验组记录.xlsx, 04_运行原始记录, columns R–T; input/material_destinations.csv	Retained workbook totals; no complete collection, tare or dry-mass ledger.	Stream-total descriptions and sand-continuity screen; no component-resolved reconstruction. Use: Describe collected-material destinations only.
Reference-washing procedure	Nine DRYWET-*-WET rows in source run workbook and S1a_Cleaning; reported protocol in Methods 2.3.	Run table retained; no energy or intensity acquisition record.	Duration-matched 600 s laboratory comparisons. Use: Laboratory stirred-water reference distinct from terminal ultrasonic washing.
Film thickness	S1_Geometry and R1_Material_Record; reported values PE 30, PP 32 and PET 25 μm.	No individual thickness readings, specimen IDs or measurement dates retained.	Nominal-geometry diagnostics using reported thickness and 30 ± 2 mm cutting target. Use: Material descriptors, with material-specific evidence restrictions.
SEM images	Raw_SEM_Records/1-PE1- M.tif, 2-PE2-M.tif, 3-PE3-M.tif and 4-PE4-M.tif.	Four native TIFF fields with retained pixels, scale bars and microscope metadata.	Full-frame four-state plate; no image-derived cleaning score. Use: Local morphology description: one analyzed field per state.
EDS spectra and reports	Raw_SEM_Records/1-PE1-M.txt through 4-PE4-M.txt (EMSA spectra) and corresponding .docx reports.	Retained EMSA spectral files and reports; metadata available within those files.	Tabulated reported fractions and eight-element normalized summaries. Use: Local composition descriptions; report sigma distinguished from specimen SD.
27-film optical repeat-reading dataset	Optical_Validation/Optical_Validation_Raw_SamePosition.csv and Optical_Validation_Raw_Reposition.csv; S2_Optical_Validation.	540 retained CSV readings; acquisition timestamp and operator fields blank; no dated instrument export.	Ten same-position and ten reposition readings per film; within-film SD summaries. Use: Separate within-film reading/repositioning variability only.
12 PE clean-film blank records	Blank_Validation/Blank_Mass_Validation_Raw.csv; S5_Blank_Validation.	Retained CSV masses; no dates, material-batch identifiers or matched cleaning Run_IDs.	Pre-terminal, terminal-washing and total changes; PE 120 s scale −1.7 to −1.1 mg and 0.82–1.49 percentage points for 13 eligible records. Use: Separate stage-specific PE sensitivity dataset; no universal subtraction.
36 waste-PE terminal-wash ratios	S4_Real_PE and input/legacy_pe.csv: 12 source batches, three ratios per batch.	Retained derived ratios; paired original m_2/m_b masses not available.	Batch means and within-batch SD from the 36 retained ratios. Use: Separate descriptive waste-PE data outside the 84 model-film runs.
Control-target example programs	Control_Logic/FB_SineAirflow.st, sine_target.py, paper1_presets.json and README.md.	Illustrative/reconstructed source code, not a retained historical controller program or measured waveform.	Sinusoidal target functions from stated parameters. Use: Explain nominal target settings only.

Dataset	Retained file and locator	Instrument-native evidence	Derived quantities and use
Earlier-study relationship	Previous_Work_Crosswalk/Previous_Work_Crosswalk.xlsx; Jin et al., DOI 10.1007/s10163-024-02111-5, pp. 305–315 and supplement.	Published article/SI support method comparisons; earlier run-level source records absent.	Apparatus/condition comparison and algebraic endpoint-equivalence checks. Use: Separate inherited apparatus/methods, retained current records, derived analyses, validation series and figures.
Sampling and weighing procedures	Source run/optical tables; S0_Provenance; retrospective procedure descriptions in MEASUREMENT_PROVENANCE.md.	No contemporaneous sampling list, fragment-level audit or full weighing-scope log.	Count-based eligibility and optical population definitions. Use: State the analyzed populations; retain conditional sampling and run-level uncertainty.

Part B follows the same dataset order. The complete eight-column table is provided in Supplementary_Data.xlsx, R1_Provenance.

Table S19 (continued). Part B: retrospective confirmations and evidence limits.

Dataset	Retrospective author confirmation	Not independently verifiable	Consequence for interpretation
84 model-film mass and count records	Authors identify a separate series and define m_0/m_1 as input films and m_2/m_b as recovered/weighed films.	Complete acquisition dates, preparation-batch history, calibration ledger and comparison with earlier run-level records.	Numerical agreement and count matching do not independently verify acquisition history or polymer-mass conservation.
2268 single-film optical readings	Mixing, selecting nine films without replacement and measuring after drying are retrospectively reported; films were not tracked across states.	Sampling list, selection probabilities, state-specific cooling/equilibration logs, zero-reference and calibration history.	Intervals omit unrecorded selection effects, shared calibration/drift and preparation dependence.
Operating conditions and sand-history labels	Controller parameter selection and old-sand heating at 600 °C for 5 h are retrospectively reported.	Sand-mass basis, transfers, additions/returns, drying/tare history and chronological batch continuity cannot be reconciled from available records.	No cumulative-use, heating-effect or regeneration inference; no independent-replicate interval for these source-labelled records.
Collected-material masses	Dry, tare-corrected and non-overlapping collections are reported retrospectively.	Recovered-sand correction, component composition, collection boundaries and transfers, including the two sand-mass jumps.	Do not close a polymer/contaminant/sand balance or substitute a total for missing-film mass.
Reference-washing procedure	Approximately 3 g film, 50 mL fresh water, 50 rpm and no additional rinse are retrospectively confirmed.	Detailed contemporaneous transfer, recovery and operating logs; energy equivalence.	No industrial-route or energy-matched superiority claim.
Film thickness	PE and PP original-lot center-point remeasurement and GREENER digital outside micrometer are reported retrospectively.	PET original experimental-lot linkage; thickness distributions and traceable calibration.	PET 25 μm is not verified thickness of the tested lot; comparisons are not thickness matched or intrinsic-polymer rankings.

Dataset	Retrospective author confirmation	Not independently verifiable	Consequence for interpretation
SEM images	Mechanical clamping/gold coating and existence of additional fields are retrospectively reported.	Specimen-to-Run_ID links, random field-selection procedure and unprovided additional fields.	No independent route-level microscopy replication or whole-sample cleanliness estimate.
EDS spectra and reports	Third-party measurement is retrospectively reported.	Full quantification software/algorithm settings, Au treatment, substrate correction and cleaning-run links.	Omission of Au does not prove coating correction; no whole-film purity or route ranking.
27-film optical repeat-reading dataset	Study-experimenter attribution and same-day timing were retrospectively reported; these do not establish campaign contemporaneity.	Actual timing relative to cleaning runs, specimen-to-Run_ID links and historical zero/calibration conditions.	Timing relative to the cleaning campaign is not independently established. Within-film variability only; no additional cleaning-run replication.
12 PE clean-film blank records	Concurrent, same-batch and condition-matched operation is retrospective author confirmation only.	Run/batch pairing, acquisition chronology, stage-transfer details and justification for transfer to contaminated films.	Not verified concurrent batch-matched correction; no PE extrapolation to PP/PET/MIX or terminal-to-pre-terminal correction.
36 waste-PE terminal-wash ratios	No additional batch-level provenance confirmation provided.	Acquisition dates, source-batch histories and paired mass records.	No reverse reconstruction of paired masses or use as independent validation of the model-film campaign.
Control-target example programs	Parameter selection via the integrated controller is retrospectively reported.	Exact historical program/model, waveform delivery, frequency/amplitude calibration and mapping to supplied example code.	Code execution does not establish that the historical apparatus executed this implementation.
Earlier-study relationship	Authors identify current records as a separate experimental series.	Direct record-to-record matching, complete chronology and independence from earlier records.	Dataset independence remains author attestation; current source links do not prove no historical run reuse.
Sampling and weighing procedures	Input/recovered weighing scopes, mixed selection without replacement, post-drying measurements and three VAL-COMP loading/treatment/recovery operations are retrospectively reported.	Selection probabilities/bias, exclusion of difficult films, cooling/equilibration, fine-fragment loss and shared-sand/order effects.	Yes flags identify retained fields/declared scope, not calibrated instruments or independently verified acquisition, batch independence or absence of loss.

Retained numerical records support recalculation but do not by themselves verify acquisition history. Retrospective author confirmations are distinguished from contemporaneous records. Blank entries or NR/NV entries do not imply that an unrecorded procedure was performed or verified. Derived quantities include recalculated endpoints and sensitivity analyses; they are not new experimental measurements.

Table S20. Reported materials, equipment and evidence limits.

Material or equipment	Reported specification and use	Evidence source and status	Unavailable information and interpretation limits
PE film	Commercial transparent film purchased through an e-commerce platform; reported center-point thickness 30 µm. 30 ± 2 mm square-side cutting target; model-film experiments.	Retained material description and retrospective author confirmation of original-lot remeasurement.	Seller/manufacturer, grade, film-forming method, molecular-weight distribution and additives unavailable. Individual thickness readings, dates and specimen IDs not retained; no thickness-matched design.
PP film	Commercial transparent film purchased through an e-commerce platform; reported center-point thickness 32 µm. 30 ± 2 mm square-side cutting target; model-film experiments.	Retained material description and retrospective author confirmation of original-lot remeasurement.	Seller/manufacturer, grade, film-forming method, molecular-weight distribution and additives unavailable. Individual thickness readings, dates and specimen IDs not retained; no thickness-matched design.
PET film	Commercial transparent film purchased through an e-commerce platform; reported center-point thickness 25 µm. 30 ± 2 mm square-side cutting target; model-film experiments. The reported 25 µm value cannot be linked to the original experimental lot.	Reported material descriptor; linkage of the 25 µm measurement to the tested lot is not established.	Original experimental-lot linkage not established; 25 µm is not a verified thickness of the tested lot. Individual readings, dates, specimen IDs, grade, production method, molecular-weight distribution and additives unavailable.
Film mixture (MIX)	Equal masses of the PE, PP and PET films listed above. Constituent thicknesses and cutting target as above; mixed-film configuration.	Retained equal-mass preparation description; inherits the evidence limits of all three constituent films.	Equal mass does not imply equal area. No unified MIX thickness is assigned; constituent-lot and thickness limitations remain.
Soil	Tongji University campus soil, dried at 60 °C for 48 h. Used in the contamination preparation described in Methods 2.2.	Retained preparation protocol; source description retrospectively confirmed by the experimenter.	Exact collection position/date, sieve size and detailed composition not retained.
Edible oil	Edible oil; film-to-oil charge mass ratio 1:0.25. Used for oil–dust preparation.	Retained preparation protocol; edible-oil description retrospectively confirmed.	Oil type, manufacturer/brand, batch identifier and composition not retained.
Quartz sand	40–70 mesh; nominal initial loading 2 kg. Cleaning medium; recorded sand masses are in S1b_Conditions.	Retained protocol and source sand-mass column; transfer-history limitation described in Section S3.	Supplier/composition and complete tare, dry-mass and transfer ledger unavailable. Retained masses do not establish one continuous sand batch; do not infer regeneration (Section S3).
Transmittance meter	LS116, new version; Shenzhen Linshang Technology Co., Ltd., Shenzhen, China; display step 0.01 percentage point over 10–100%. Single-film center readings; fixed-position and repositioning measurements (Sections S6 and S7).	Retained Methods and retrospective model/version identification; specified display step is an instrument specification.	Calibration, verification and zero/reference records unavailable. Display resolution is not absolute accuracy.

Material or equipment	Reported specification and use	Evidence source and status	Unavailable information and interpretation limits
Analytical balance	AL204-IC; METTLER TOLEDO; readability 0.0001 g (0.1 mg). One stable net reading after taring.	Retained Methods identify the instrument; single stable net reading after taring is retrospective author confirmation.	Calibration certificates and contemporaneous check-mass records unavailable. Readability is not a calibration error or uncertainty bound.
Ultrasonic cleaner	KQ3200DE; Kunshan Ultrasonic Instruments Co., Ltd. (Shumei), Kunshan, China; 40 kHz; 150 W nameplate input; 100% setting. Terminal-washing procedure TW-01 (Table S10).	Retained TW-01 protocol reports the instrument and nameplate/settings values; no independently measured liquid acoustic power.	Actual liquid temperature history and delivered acoustic power unavailable; nameplate input is not measured liquid power.
Scanning electron microscope	ZEISS Sigma 300; serial Sigma 300-8211011409; SE2 detector; SEM software V07.00.00.00. Native TIFF settings: 1000×, 15 kV and 10 μm scale bars; four supplied treatment-state fields (Section S8).	Native TIFF headers identify model, serial, detector and software; image bars supply settings. ZEISS identity checked against manufacturer documentation.	Cleaning-Run_ID and preparation-batch links, field-selection records and additional fields unavailable for analysis.
Outside micrometer	GREENER digital outside micrometer; 0–25 mm range; 0.001 mm (1 μm) display resolution. Reported thickness measurements at film centers.	Retained reported identification and retrospective author confirmation; no individual thickness-reading export.	Complete manufacturer/model designation and calibration record not retained. Display resolution does not establish accuracy; individual readings and measurement dates are unavailable.
Blower	Manufacturer/model not retained. Controlled gas supply. Gas–solid cleaning apparatus (Section S3).	Retained apparatus description; this description is not an instrument nameplate or model record.	Manufacturer/model not retained; historical operating trace unavailable.
Variable-frequency drive	Manufacturer/model not retained. Variable-frequency gas-supply control. Operating parameters selected through the integrated controller.	Retained apparatus description and retrospective explanation of parameter selection.	Manufacturer/model not retained; contemporaneous controller export/settings archive unavailable.
Control cabinet/controller	Manufacturer/model not retained. Integrated controller with a built-in program. Selection of operating parameters for the reported cleaning conditions.	Retrospective author confirmation of parameter selection through the built-in program.	Manufacturer/model not retained; no recovered firmware/program export or measured instantaneous gas-flow waveform.
Gas-velocity meter	Manufacturer/model not retained. Recorded mean gas-velocity values. Recorded values are provided in S1b_Conditions.	Source workbook contains mean-velocity readings; no instrument-native continuous trace.	Manufacturer/model and calibration record not retained; the recorded mean is not an instantaneous waveform.
Humidity meter	Manufacturer/model not retained. Nominal inlet relative-humidity settings. Nominal RH configurations described in Section S3.	Retained nominal RH labels and environmental-method description; no continuous inlet record.	Manufacturer/model and calibration record not retained. Nominal RH does not establish actual film/sand moisture exposure.

Material or equipment	Reported specification and use	Evidence source and status	Unavailable information and interpretation limits
Drying oven	Manufacturer/model not retained. Drying protocols at 45–50 °C, 50 °C and 60 °C. Contamination preparation, post-treatment drying and terminal-wash drying (Methods 2.2; Sections S3 and S10).	Retained preparation/drying protocols; no temperature-time instrument log.	Manufacturer/model and calibration record not retained; stated protocols do not verify every recorded run's temperature history.
Humidifier	Manufacturer/model not retained. Evaporative humidity adjustment. Establishment of the nominal environmental configurations.	Retained environmental-method description.	Manufacturer/model not retained; no continuous exposure record.
Dehumidifier, air conditioner and circulation fans	Manufacturer/model not retained. Environmental adjustment and air circulation. Establishment of the nominal environmental configurations.	Retained environmental-method description.	Manufacturer/model not retained; no continuous exposure record.
Mechanical stirrer	Manufacturer/model not retained. 50 $r \cdot min^{-1}$. Laboratory stirred-water reference (Section S3).	Retained stirred-water reference protocol.	Manufacturer/model not retained; no measured mixing power or energy input.
Muffle furnace	Manufacturer/model not retained. Reported sand heat-treatment setting: 600 °C for 5 h. Source labels identify ten BOUND-OIL-CYCLE records and three BOUND-OIL-RESTORE records; they do not verify run-to-run sand continuity.	Source labels and retrospective report of 600 °C for 5 h; sand continuity and thermal exposure are not independently established.	Manufacturer/model not retained. The unresolved sand-mass/transfer history precludes a verified continuous-use or heating-effect interpretation.
EDS acquisition/quantification system	Third-party analysis; four EMSA/MAS spectra and four eight-element reports. Spectrum-header magnification: 1000 for PE1–PE3 and 1169 for PE4; local elemental analysis (Section S8).	Four native EMSA/MAS files and four reports; third-party EDS acquisition is retrospective author confirmation.	Manufacturer/model, laboratory identity, quantification software/version, algorithm, standardization and Au treatment not retained; local normalized output only.

Reported instrument specifications and retrospective author confirmations are distinguished from native measurement files. Missing identifiers are not inferred from a device's stated function. Display resolution and balance readability are not measurement-uncertainty estimates.

Table S21. Group-level gravimetric eligibility and within-group selection description.

Group	Total *n*	Eligible *n*	Excluded *n*	Eligible (%)
BBD	17	6	11	35.3
VAL-HC	3	0	3	0.0
VAL-LE	3	2	1	66.7
VAL-COMP	3	0	3	0.0
MAT-PP-COMP	3	1	2	33.3
MAT-PET-COMP	3	1	2	33.3
MAT-MIX-COMP	3	2	1	66.7
CTRL-CF-LOW	3	2	1	66.7

Group	Total n	Eligible n	Excluded n	Eligible (%)
CTRL-CF-MID	3	3	0	100.0
CTRL-CF-HIGH	3	0	3	0.0
BOUND-HUM-60	3	0	3	0.0
BOUND-HUM-80	3	0	3	0.0
BOUND-LOAD-HIGH	3	0	3	0.0
BOUND-OIL-CYCLE	10	0	10	0.0
BOUND-OIL-RESTORE	3	0	3	0.0
DRYWET-PE-DRY	3	0	3	0.0
DRYWET-PE-WET	3	2	1	66.7
DRYWET-PP-DRY	3	1	2	33.3
DRYWET-PP-WET	3	3	0	100.0
DRYWET-PET-DRY	3	0	3	0.0
DRYWET-PET-WET	3	3	0	100.0

Group, total/eligible/excluded n and eligibility percentage; within-group optical and count summaries by eligibility.

All 21 group eligibility rows are shown. Full within-group comparisons are electronic; no pooled comparison is interpreted as removing selection bias.

Electronic source: R1_Eligibility; Eligibility_Groups.csv, Selection_Description.csv

Table S22. Clean-film omission scenarios for incomplete-recovery runs.

Run	Mass factor α	Scenario missing clean mass (mg)	Scenario contribution (pp)	Scenario apparent index (%)
BBD-R02	0.5	12.507	9.7	89.1
BBD-R02	1.0	25.014	19.5	79.4
BBD-R02	1.5	37.521	29.2	69.6

Run_ID, omitted piece count, mean clean-film mass, α, scenario q, unrestricted index and scenario index/shift.

The three BBD-R02 scenarios are shown here; all 174 rows (58 runs × three multipliers) are in the electronic table. Scenario fields never replace measured masses.

Electronic source: R1_Missing_Film; Missing_Film_Scenarios.csv

Table S23. State-level optical variation and run-level conditional sensitivity.

Quantity	Population	Range or maximum	Unit
Denominator $\overline{T}_0 - \overline{T}_1$	84 runs	68.01–76.25	percentage points
Conditional standard uncertainty of I_t	84 runs	0.33–0.99	percentage points
Denominator / sampling uncertainty	84 runs	98.79–241.40	dimensionless
Maximum display-rounding change	84 runs	0.01470	percentage points
Clean-state film SD	84 states	0.455–1.790	percentage points

Quantity	Population	Range or maximum	Unit
Contaminated-state film SD	84 states	0.458–1.661	percentage points
Post-cleaning-state film SD	84 states	0.230–1.592	percentage points

252 state means/SDs/SEs/ranges; 84 denominators, propagated sampling components, $D/u_{\mathrm{sampling}}(D)$, resampling limits and rounding sensitivities.

Ranges summarize the state SDs and run sensitivities; all 252 state and 84 run rows are in the electronic tables. Position-validation variance is not double-counted; unrecorded systematic and selection effects are not included.

Electronic source: R1_Optical_Uncertainty, S2_State_Summary; Optical_State_Summary.csv, Optical_Uncertainty.csv

Table S24. Endpoint-specific conditional group intervals and five optical contrasts.

Optical contrast (B – A)	n A / B	Difference (pp)	Conditional 95% interval (pp)
PE: water minus dry	3 / 3	1.1	0.5 to 1.7
PP: water minus dry	3 / 3	2.2	0.8 to 3.6
PET: water minus dry	3 / 3	1.9	1.4 to 2.4
Nominal 80 minus 60% RH	3 / 3	-12.2	-14.9 to -9.5
Nominal 5 minus 3 g	3 / 3	-15.2	-18.9 to -11.5

Endpoint-specific n, mean, SD and conditional t limits; five directed optical contrasts with SE, Welch degrees of freedom and limits.

All five Welch contrasts are shown; full endpoint-specific group summaries are in the electronic table. BBD and the source-labelled sand-history records remain descriptive; no interval is assigned to one eligible value or an excluded endpoint.

Electronic source: R1_Group_CI, R1_Contrasts; Group_Conditional_CI.csv, Optical_Contrasts.csv

Table S25. Stage-specific PE blank responses and their scale relative to eligible PE 120 s runs.

PE blank	Protocol	Pre-terminal change (mg)	Terminal change (mg)	Total change (mg)
BLK-PE-A-01	drying-only	-1.2	—	-1.4
BLK-PE-A-02	drying-only	-1.5	—	-1.1
BLK-PE-A-03	drying-only	-0.9	—	-1.0
BLK-PE-B-01	terminal-wash-only	—	-1.8	-1.9
BLK-PE-B-02	terminal-wash-only	—	-2.5	-2.6
BLK-PE-B-03	terminal-wash-only	—	-2.0	-2.1
BLK-PE-C-01	120-s-gas-solid-plus-terminal-wash	-1.4	-2.3	-3.8
BLK-PE-C-02	120-s-gas-solid-plus-terminal-wash	-1.1	-2.9	-4.0
BLK-PE-C-03	120-s-gas-solid-plus-terminal-wash	-1.7	-2.9	-4.6
BLK-PE-D-01	600-s-gas-solid-plus-terminal-wash	-1.8	-3.5	-5.3

PE blank	Protocol	Pre-terminal change (mg)	Terminal change (mg)	Total change (mg)
BLK-PE-D-02	600-s-gas-solid-plus-terminal-wash	-1.5	-4.2	-5.6
BLK-PE-D-03	600-s-gas-solid-plus-terminal-wash	-2.0	-3.4	-5.3

All 12 blank-stage records with original missing cells; b_{pre}, $b_{terminal}$, b_{total} and stage discrepancies; scale scenarios for 13 eligible PE 120 s observations.

The tables report all 12 record-stage values and 13 run-specific scales. Total post-terminal losses do not correct the pre-terminal endpoint; PE responses are not transferred to other materials.

Electronic source: R1_Blank_Scale; Blank_Stage_Changes.csv, Blank_Stage_Summary.csv, Blank_Scale.csv

Table S25 (continued). Pre-terminal blank scale for 13 eligible PE 120 s runs.

Run	Contamination increment (mg)	Minimum scale (pp)	Maximum scale (pp)
BBD-R01	123.6	0.89	1.38
BBD-R03	134.4	0.82	1.26
BBD-R05	114.1	0.96	1.49
BBD-R12	121.0	0.91	1.40
BBD-R13	125.9	0.87	1.35
BBD-R16	121.3	0.91	1.40
VAL-LE-02	119.7	0.92	1.42
VAL-LE-03	132.3	0.83	1.28
CTRL-CF-LOW-02	122.5	0.90	1.39
CTRL-CF-LOW-03	120.7	0.91	1.41
CTRL-CF-MID-01	124.8	0.88	1.36
CTRL-CF-MID-02	129.7	0.85	1.31
CTRL-CF-MID-03	114.1	0.96	1.49

The scale uses absolute C-group pre-terminal changes of 1.1–1.7 mg divided by each apparent contamination increment. These are stage-specific PE sensitivity values: acquisition dates, batch identifiers and matched cleaning Run_IDs are unavailable. They are not subtracted from primary observations or transferred to PP, PET or MIX.

Supplementary figures

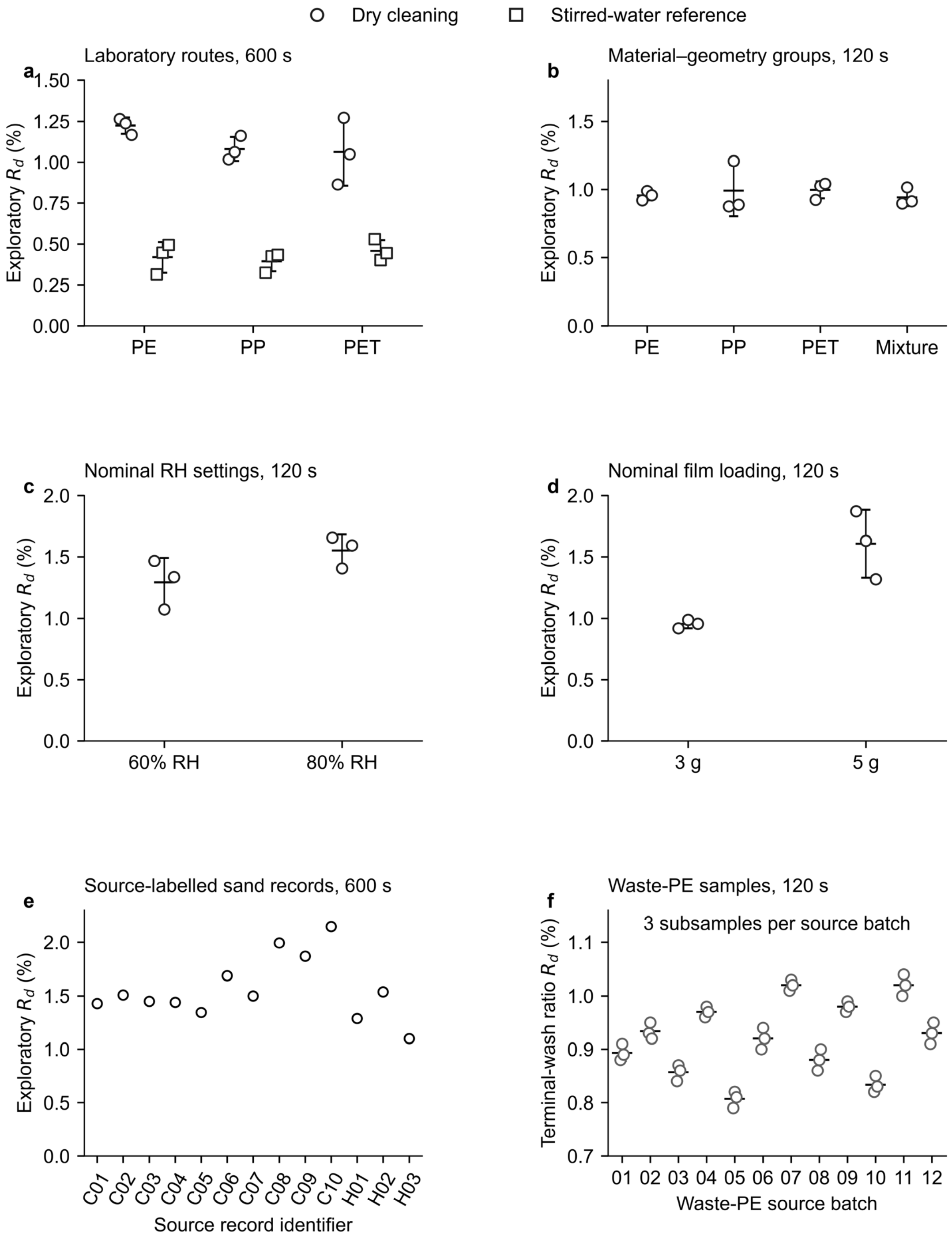

Figure S1. Operational terminal-wash mass-change ratio, R_d. (a–d) Three recorded cleaning runs per model-film group; points show runs, horizontal lines means and bars sample SDs. Paired masses refer to the same recovered films across the distinct reported drying procedures in Section S10. (e) Unconnected points for the 13 records labelled BOUND-OIL-CYCLE and BOUND-OIL-RESTORE. C01–C10 and H01–H03 identify source records, not a verified use chronology; sand-transfer history and batch continuity are not independently established. The points do not estimate cumulative-use or heating effects. (f) All 36 waste-PE terminal-wash ratios in 12 batches with three subsamples each and within-batch means ± SD. R_d is an operational mass response, not a purity or contaminant-removal measurement.

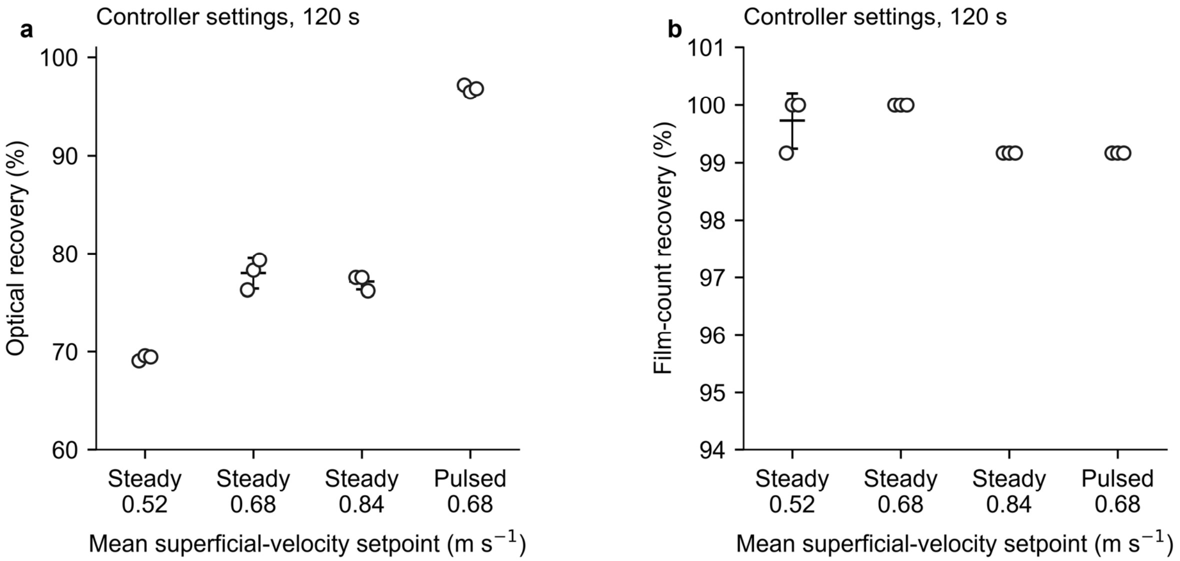

Figure S2. Optical recovery, I_t, and film-count recovery, R_N, under constant-flow and pulsed-flow settings, with three recorded runs per group. Points, horizontal lines and bars show runs, means and sample SDs. The horizontal axis gives nominal mean-velocity settings; pulsed operation uses nominal amplitude 0.12 m·s^{-1} and frequency 1.50 Hz. No instantaneous waveform is reconstructed. Conditional run-level intervals are in Table S24. VAL-COMP is reused where indicated and counted once.

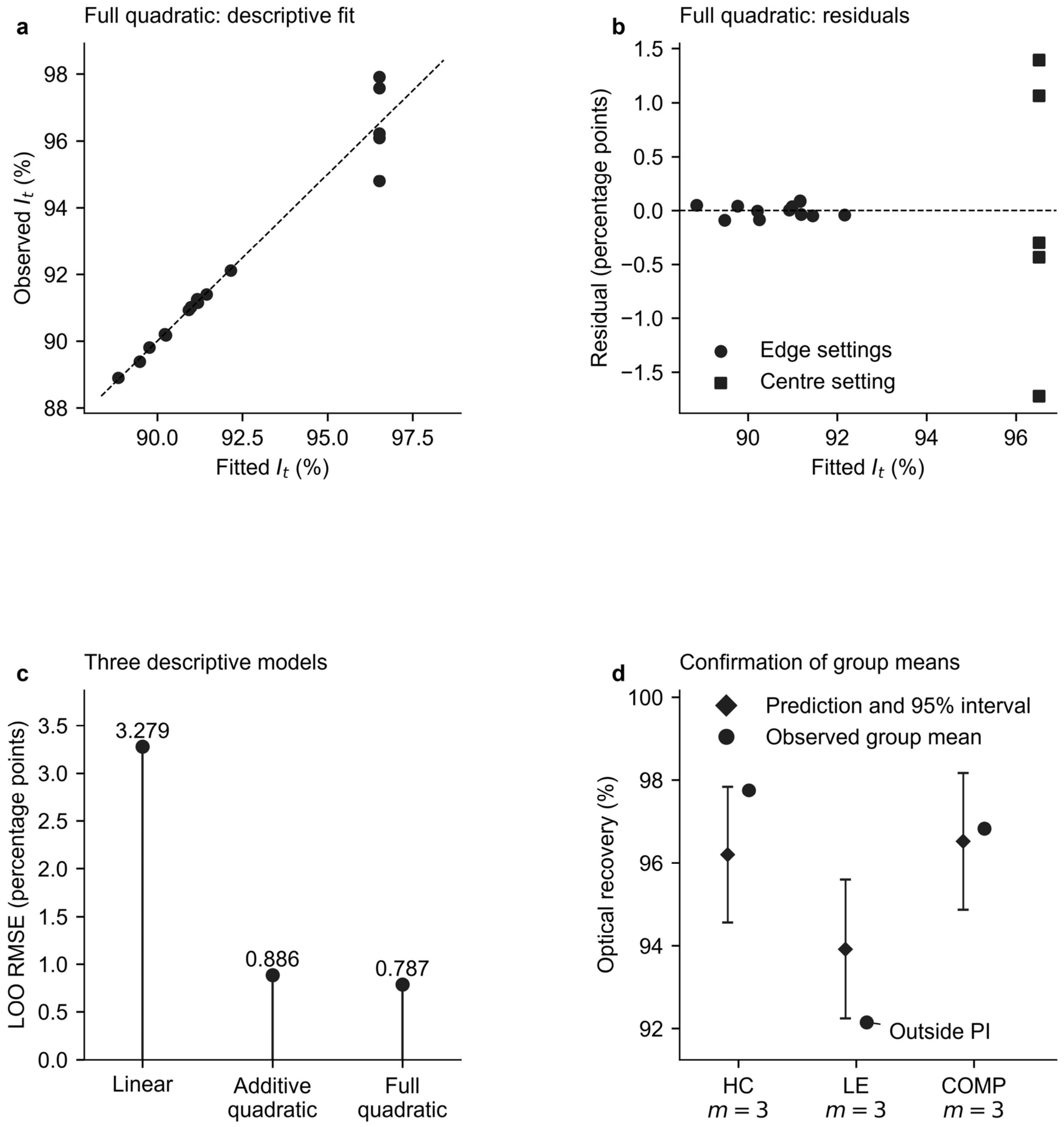

Figure S3. Optical-model comparison and full quadratic diagnostics. (a) Observed and fitted I_t for 17 design runs; dashed line, identity. (b) Full quadratic residuals versus fitted values. (c) Leave-one-out RMSE for the linear, additive quadratic, and full quadratic models fitted to the same observations. (d) Observed confirmation means (three runs per group) and full quadratic 95% prediction intervals for means of three future runs. Confirmation runs were excluded from fitting. VAL-LE lies below its group-mean interval. Intervals are conditional on the error assumptions; the fitted surface is descriptive.

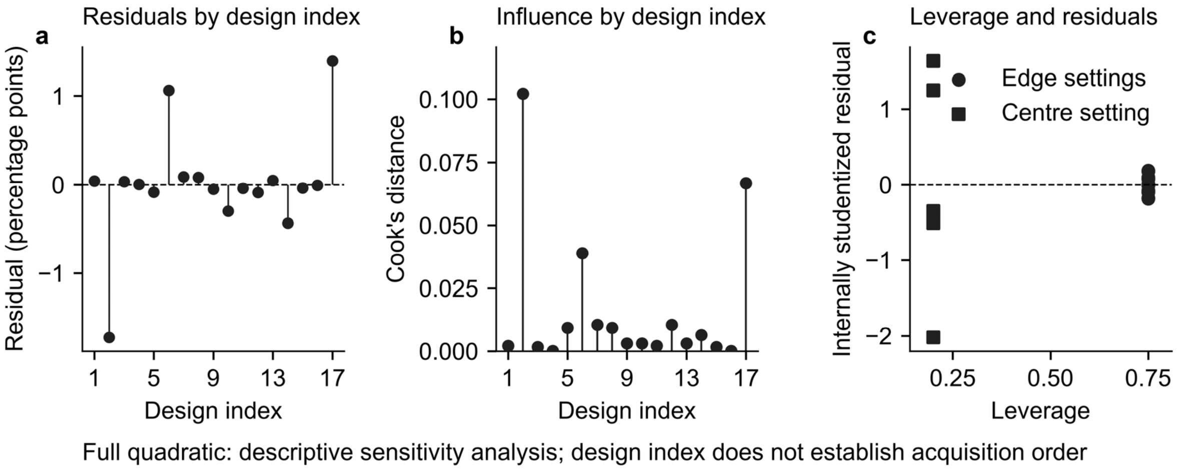

Figure S4. Design-identifier and influence diagnostics for the full quadratic I_t model (17 Box–Behnken observations). (a) Residuals by design identifier; dashed line, zero. Identifiers do not establish actual execution order, so this panel does not assess temporal drift. (b) Cook's distance. (c) Leverage versus internally studentized residuals, distinguishing center and noncenter settings. No observations were excluded on the basis of these diagnostics.
